# Genetic Structure of Blue Ling, *Molva dypterygia*, in the North Atlantic

**DOI:** 10.1002/ece3.72801

**Published:** 2026-03-27

**Authors:** María Quintela, Hege Øverbø Hansen, Laila Unneland, Ilaria Coscia, Torild Johansen, Lise Helen Ofstad, Kristin Helle

**Affiliations:** ^1^ Institute of Marine Research (IMR) Bergen Norway; ^2^ Foras na Mara – Marine Institute Oranmore Co. Galway Ireland; ^3^ Institute of Marine Research (IMR) Tromsø Norway; ^4^ Faroe Marine Research Institute (FAMRI) Tórshavn Faroe Islands

**Keywords:** blue ling, conservation genetics, *Molva dypterygia*, population structure, SNP, stock

## Abstract

The blue ling, a deep‐water fish widespread in the Northeast Atlantic, has suffered major population declines from intensive fishing since the 1970s. Individuals sampled from four Norwegian fjords and offshore locations, including the Norwegian shelf, Faroe Islands, Rockall, Iceland and Greenland, were genotyped at 61 SNP loci. Results revealed weak but significant overall differentiation (*F*_ST_ *=* 0.005***) and no evidence of isolation by distance. While fjord and offshore groups showed no broad genetic separation, Yrkefjord displayed a distinct pattern relative to most other locations, warranting further investigation. Moreover, linkage disequilibrium analysis of SNPs produced a PCA pattern consistent with the characteristic three‐band structure associated with chromosomal inversions.

## Graphical abstract

The blue ling, a deep‐water fish in the Northeast Atlantic, has experienced severe population declines since the 1970s due to intensive fishing. Genetic analysis across fjords and offshore regions using 61 SNPs revealed weak but significant differentiation, rejecting the idea of a single randomly mating population. While no clear split was found between fjord and offshore groups, notable genetic differences emerged in Yrkefjorden compared to other locations, suggesting localised structure worth further study.

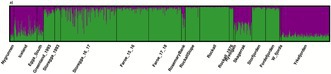

## 1 Introduction

The blue ling, *Molva dypterygia* (Pennant 1784) is a benthopelagic gadoid fish belonging to the family *Lotidae* (cuskfishes, burbots, hakes) (Froese and Pauly 2023). In the NE Atlantic, the species is distributed from the Barents Sea along the coast of Norway to the west of the British Isles, around the Faroe Islands and Iceland and off the east coast of Greenland (Large et al. 2010). Reports of blue ling further south (typically in the Bay of Biscay) are due to the confusion with the Spanish ling, *Molva macrophthalma* , a more southerly‐distributed species extending to Morocco and into the Mediterranean and formerly considered a sub‐species of blue ling (Priede 2019). Meristic features differ between blue ling ( *M. dypterygia* ) and the Spanish ling ( *M. macrophthalma* ) as the pelvic fin extends beyond the pectoral fin in the latter.

Blue ling displays aggregating spawning behaviour, and high densities of individuals can be found in deep waters in five areas off North‐western Scotland: i.e., (1) along the continental northwest slope; (2) on, around and northwest of Rosemary Bank; (3) on the southern and southwest margins of Lousy Bank; (4) on the northeast margins of Hatton Bank; (5) on the eastern and southern margins of Hatton Bank mainly at depths of 730–1100 m (Ehrich 1982; Hislop et al. 2015; Large et al. 2010), as well as in parts of the northern North Sea (Wheeler 1969), Rockall (Ehrich 1982), west of the Hebrides, Reykjanes bank south of Iceland, around the Faroe Islands and along Storegga (Large et al. 2010; Magnússon et al. 1997). As typical for gadoid fishes, fecundity is high: from 1 to 3.5 million eggs per female (Gordon and Hunter 1994; Thomas 1987), and spawning occurs from February to June in the area west of Scotland (Large et al. 2010), from April to May in the North Atlantic (Cohen et al. 1990), in May–June along Storegga (Engås 1983; Magnússon et al. 1997; Thomas 1987) and from March–April to August in the Norwegian Deep (Bergstad 1991). The information about larval dispersal and nursery areas for early‐stage demersal juveniles is scarce because ichthyoplankton surveys generally target more inshore waters, therefore eluding the species.

Commercial trawl and longline fisheries now target adult blue ling aged 9–19 years (ICES 2018) on the western slopes of Scotland, around the Faroe Islands and Iceland. The spawning aggregating behaviour of blue ling has historically attracted intense fishing pressure, particularly through gillnet fisheries along the Norwegian coast in the 1970s and 1980s, which led to the depletion of the stock in the region. In response, the species was included in the Norwegian Red List in 2006 (Kålås et al. 2006), and in 2009, two measures were introduced in Norway to promote the stock recovery: a ban on targeted fishing and a bycatch limit of 10% of the total catch. Consequently, no TAC (Total Allowable Catch) is set for blue ling in Norwegian waters, and current ICES advice recommends a zero‐catch policy under a precautionary approach. The bycatch restriction effectively limited fishing activity on the spawning aggregations and at the same time imposed significant constraints on fisheries targeting common ling ( *Molva molva* ) and tusk ( *Brosme brosme* ).

Sustainable fisheries management requires alignment between biologically relevant processes and regulatory actions. Thus, understanding genetic population structure and connectivity is critical for identifying appropriate management units (e.g., Cadrin et al. 2014; Reiss et al. 2009). In recent years, this has been aided by the discovery and use of loci exhibiting signatures of selection, which have proven effective for detecting fine‐scale spatial genetic structure and delineating biologically meaningful units for fisheries management (Andersson et al. 2024). Failure to account for key factors—such as the spatio‐temporal mixing of populations or inadequate identification of locally‐adapted populations, which are particularly vulnerable to overfishing (Funk et al. 2012; Pinsky and Palumbi 2014; Waples et al. 2008) − can lead to potential overexploitation of resources (Allendorf et al. 2008; Kerr et al. 2017). In blue ling, reproductive behaviour has been thought to dilute signals of spatial genetic differentiation; however, genotyping‐by‐sequencing (GBS) analyses revealed the presence of a strong phylogeographic break separating the Norwegian coast and offshore Atlantic populations (McGill et al. 2023). Despite these findings, information on how the genetic populations interact between fjords and oceanic areas as well as differences among Norwegian fjords is lacking. Significant genetic differentiation has been documented between offshore and fjord populations in Atlantic cod (Johansen et al. 2020; Jorde et al. 2021), however, McGill et al. (2023) were unable to draw similar conclusions for blue ling, largely due to limited sampling coverage. If there are separate and genetically different populations in the fjords, they might be more vulnerable to overfishing and depletion as well as more sensitive to environmental and climate changes as their genetic pool would be smaller (Reiss et al. 2009). On the other hand, they might support a local fishery that, on its small scale, would be sustainable.

The aim of this study was to assess patterns of genetic differentiation between coastal/fjord samples and offshore blue ling populations and, by expanding the sampling in Norwegian fjords, to further unravel potential genetic differences within these areas. Blue ling individuals were genotyped using an available panel of species‐specific SNP (Single Nucleotide Polymorphism) markers (Helle et al. 2020). Both newly collected and historical samples were analysed to evaluate the temporal stability of any genetic patterns identified in this study. The findings of this study will contribute to a deeper understanding of the evolutionary processes at work in the deeper layers of the oceans, as well as produce information that will be useful in refining the approach to management of blue ling.

## 2 Materials and Methods

### 2.1 Sampling and Genotyping

**FIGURE 1:**
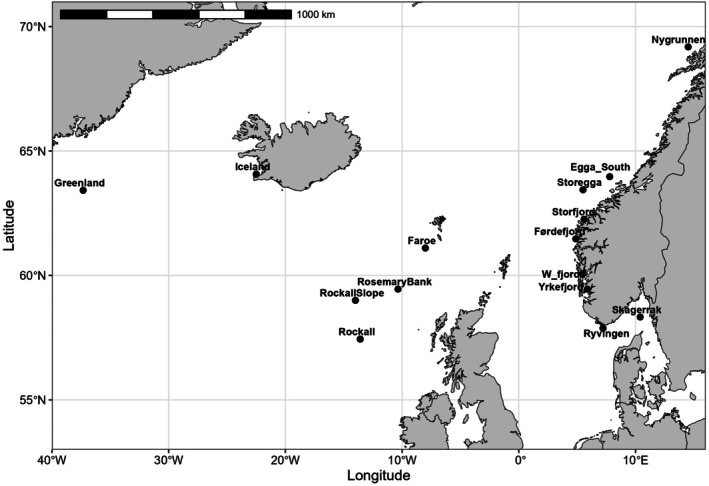
*Molva dypterygia* sampling sites. The map was created using the geom_sf() function in the R package *ggplot2* (Wickham 2016).

A total of 1433 individuals was collected in 15 different locations (Figure 1) using several approaches: (1) on board of research vessels during scientific surveys, (2) by professional fishers, especially the Norwegian Reference Fleet and (3) by sport anglers (Table 1). The fish used in this study were sampled using standard sampling protocols to meet scientific and ethical requirements. Individuals were collected from Yrkefjorden (a fjord east of Haugesund; inner Boknafjorden), Førdefjorden, Storfjorden (near Ålesund), Storegga (Norwegian shelf at Møre, west off Ålesund), Skagerrak and near the Faroe Islands, Iceland, Rosemary Bank and Rockall. DNA was extracted from ethanol‐preserved finclips or otoliths (historical samples) using the Qiagen DNeasy Blood & Tissue Kit (QIAGEN) in 96 well‐plates. Individuals were genotyped with the suite of dedicated 81 SNP loci mined from ddRAD‐sequencing as described in Helle et al. (2020). The list of loci with their corresponding flanking regions and the distribution in multiplex reactions can be found in table 1 in Helle et al. (2020). SNPs were distributed into three multiplex assays and genotype calling was performed using the Sequenom MassARRAY iPLEX Platform as described by Gabriel et al. (2009). Briefly, a locus‐specific PCR reaction was conducted containing 0.78 μL of the 10× PCR buffer, 0.40 μL of MgCl_2_ (25 mM), 0.125 μL of a mix dNTP (25 mM), 0.625 μL of the Primer Mix (Table S1), 0.125 μL of the HotStar Taq Plus (5 U/μL), 1.35 μL of water and 2 μL of the template DNA. The PCR program was 95°C 2 min, followed by 45 cycles of 95°C 30 s, 56°C 30 s and 72°C and a final extension step of 72°C for 5 min. The PCR products were then purified by adding 2 μL of SAP cocktail (containing 0.17 μL of SAP buffer, 0.3 μL of 1.7 U/μL shrimp alkaline phosphatase and dsH_2_O) and incubated at 37°C for 30 min and 85°C for 15 min. Finally, a locus‐specific primer extension reaction was performed where the primers anneal next to the intended genotyped site in a reaction of 2 μL containing 0.22 μL 10X iPLEX buffer, 0.2 μL of the iPLEX extension mix, 1 μL of the probe mix (Table S1), 0.05 μL of the iPLEX enzyme and water. The primer extension thermal cycling was 94°C 30 s, followed by 40 cycles of 94°C 5 s and 5 cycles of 52°C 5 s and 80°C 5 s, to end with 3 min at 72°C. The resulting products were then cleaned using SpectroClean resin (Sequenom) and spotted into 384‐well sample SpectroCHIPs (Sequenom) following the manufacturers' protocol.

**TABLE 1:** Sample summary statistics obtained for the set of 60 SNP loci: Sampling sites and sampling year with geographic coordinates in decimal degrees, number of individuals (*N*), percentage of polymorphic loci, observed heterozygosity, *H*_o_ (mean ± SE), unbiased expected heterozygosity, u*H*_e_ (mean ± SE), inbreeding coefficient, *F*_IS_ (mean ± SE), number of deviations from Hardy–Weinberg equilibrium (HWE) at *α* = 0.05 both before and after False Discovery Rate (FDR) correction. Samples marked with * are considered historic samples.

Type	Area	Sample	Year	Latitude	Longitude	*N*	*H*_o_	u*H*_e_	*F*_IS_	No dev HWE (FDR)
Coastal	Greenland	Greenland*	1993	63.42	−37.33	34	0.339 ± 0.020	0.341 ± 0.018	−0.008 ± 0.023	5 (0)
	Iceland	Iceland	2017–2019	64.07	−22.50	70	0.336 ± 0.017	0.345 ± 0.016	0.027 ± 0.019	7 (0)
	West of Scotland	Rockall_1976*	1976	57.44	−13.59	13	0.359 ± 0.024	0.336 ± 0.020	−0.107 ± 0.031	1 (0)
		Rockall	2010–2011	57.44	−13.59	90	0.339 ± 0.016	0.348 ± 0.016	0.014 ± 0.014	2 (1)
		Rockall Slope	2007, 2011	59.00	−14.00	44	0.347 ± 0.020	0.345 ± 0.017	−0.002 ± 0.022	4 (0)
		Rosemary Bank	2007	59.45	−10.35	12	0.374 ± 0.021	0.364 ± 0.017	−0.084 ± 0.031	1 (0)
	Faroe Islands	Faroe_15_16	2015–2016	61.10	−8.01	98	0.350 ± 0.017	0.350 ± 0.016	−0.013 ± 0.013	2 (0)
		Faroe_16_18	2016–2018	61.05	−8.09	100	0.341 ± 0.018	0.344 ± 0.017	0.010 ± 0.017	7 (1)
	Norway	Nygrunnen	2013	69.18	14.51	12	0.334 ± 0.020	0.346 ± 0.020	−0.023 ± 0.031	1 (0)
		Egga_South	2020	63.97	7.79	20	0.368 ± 0.024	0.329 ± 0.018	−0.129 ± 0.029	3 (0)
		Storegga_1993*	1993	63.41	5.54	18	0.330 ± 0.022	0.337 ± 0.018	0.009 ± 0.037	4 (1)
		Storegga_16_17	2016–2017	63.44	5.50	171	0.344 ± 0.017	0.348 ± 0.016	0.009 ± 0.010	4 (0)
		Ryvingen	2014	57.88	7.21	5	0.354 ± 0.026	0.365 ± 0.021	−0.096 ± 0.044	1 (0)
		Skagerrak	2015–2021	58.33	10.40	51	0.334 ± 0.017	0.346 ± 0.017	0.021 ± 0.019	3 (0)
Fjord	Norway	Storfjorden	2018	62.26	5.53	42	0.346 ± 0.021	0.346 ± 0.017	0.002 ± 0.023	4 (0)
		Fordefjorden	2015, 2019	61.47	4.88	43	0.345 ± 0.020	0.343 ± 0.017	−0.011 ± 0.023	4 (0)
		W_fjords	2014	60.06	5.45	5	0.342 ± 0.030	0.340 ± 0.024	−0.116 ± 0.048	2 (0)
		Yrkefjorden	2016, 2021	59.45	5.88	146	0.347 ± 0.016	0.348 ± 0.016	−0.002 ± 0.011	6 (0)

### 2.2 Statistical Analysis

After purging loci that failed to amplify in > 15% of the individuals or in an entire sample, as well as individuals showing > 15% missing markers, a final set of 974 individuals distributed across 18 samples and genotyped at 61 polymorphic markers was retained for statistical analyses (Table 1). Five out of the 61 retained loci showed missing data ranging from 5% to 13% whereas 74% of the total loci displayed ≤ 1% missing data. Due to the historical value of the Rockall sample from 1976, a maximum of 18% missing data per individual was allowed.

To identify loci putatively under selection, two complementary approaches were employed: BayeScan 2.1 (Foll and Gaggiotti 2008) and Arlequin v.3.5.1.2 (Excoffier et al. 2005). In BayeScan, the sample size was set to 10,000 iterations with a thinning interval of 50; loci with posterior probabilities > 0.99—corresponding to a Bayes Factor > 2 and interpreted as ‘decisive selection’ (Foll and Gaggiotti 2006)—were considered candidate outliers. In Arlequin, simulations were performed under a hierarchical island model with 1000 demes and 50,000 replicates. The intersection of loci identified by both methods was used to derive a consensus set of candidate loci putatively under positive selection.

The power of the dataset was assessed with a genotype accumulation curve built using the function *genotype curve* in the R (R Core Team 2025) package *poppr* (Kamvar et al. 2014). This approach can assess if the SNP panel would accurately discriminate between individuals by randomly sampling × loci without replacement and counting the number of observed multilocus genotypes (MLGs). This was repeated *r* times for 1 locus up to n‐1 loci, creating n‐1 distributions of observed MLGs.

Conformance with gametic phase equilibrium (LD) and Hardy–Weinberg proportions (HWE) was examined for all loci and samples using GENEPOP 7 (Rousset 2008). The observed (*H*_o_) and unbiased expected heterozygosity (*uH*_e_) as well as the inbreeding coefficient (*F*_IS_) per sample were computed with GenAlEx v6.1 (Peakall and Smouse 2006).

A Principal Component Analysis (PCA) was conducted using the function *dudi.pca* in *ade4* (Dray and Dufour 2007). Supervised genetic structure using geographically explicit samples was assessed using the Analysis of Molecular Variance (AMOVA) and pairwise *F*_ST_ (Weir and Cockerham 1984), both computed with Arlequin v.3.5.1.2 (Excoffier et al. 2005) using 10,000 permutations. The differentiation driven by habitat (open coast vs. fjords) was addressed using the hierarchical approach in AMOVA. The False Discovery Rate (FDR) correction of Benjamini and Hochberg (1995) was applied to *p*‐values to control for Type I errors. The relationship among samples was also examined using the Discriminant Analysis of Principal Components (DAPC) (Jombart et al. 2010) implemented in the R (R Core Team 2025) package *adegenet* (Jombart 2008). Groups were defined using geographically explicit locations, and to avoid overfitting, both the optimal number of principal components and discriminant functions to be retained were determined through cross validation using the *xvalDapc* function (Jombart and Collins 2015; Miller et al. 2020). Likewise, the Bayesian clustering approach implemented in STRUCTURE v. 2.3.4 (Pritchard et al. 2000) was used via ParallelStructure (Besnier and Glover 2013) to identify genetic groups under a model assuming admixture and correlated allele frequencies across a number of clusters ranging from *K* = 1 to *K* = 10. Analyses were conducted both with and without using sample group information, as the former aids in detecting lower levels of divergence without biasing towards detecting structure when it is not present (Hubisz et al. 2009). STRUCTURE output was further analysed through: (a) the *ad hoc* summary statistic Δ*K* of Evanno et al. (2005) and (b) the Puechmaille (2016) four statistics (MedMedK, MedMeanK, MaxMedK and MaxMeanK) both implemented in StructureSelector (Li and Liu 2018). Finally, the 10 runs for the selected Ks were averaged with CLUMPP v.1.1.1 (Jakobsson and Rosenberg 2007) using the FullSearch algorithm and the G′ pairwise matrix similarity statistic and graphically displayed using barplots.

The relationship between genetic (*F*_ST_) and geographic distance was examined to investigate if it conformed to the expectations of an “Isolation by Distance” pattern (IBD), i.e., increasing genetic differentiation with geographic distance as a result of drift and restricted gene flow (Rousset 1997; Slatkin 1993; Wright 1943). A two‐tailed Mantel (1967) test was conducted using PASSaGE v2 (Rosenberg and Anderson 2011), and significance was assessed via 10,000 permutations. The matrix of pairwise shortest distance by water was obtained by calculating least‐cost distances via seas (avoiding landmasses) between sampling sites using the *lc.dist* function from the (R Core Team 2025) package *marmap* v1.0 (Pante and Simon‐Bouhet 2013).

Putative clines of allele frequency were investigated via the latitudinal sliding‐window approach developed by Pereira et al. (2018). To verify if the putative observed clines were due to random chance, permutation tests were conducted where the location of the populations was switched for all the alleles reported as significant (summary statistic used: slope; number of permutations per allele: 10,000). Finally, loci identified as displaying clines were subjected to a geographic cline analysis conducted using the R package HZAR (Derryberry et al. 2014) over a transect starting in Nygrunnen (69°N) and south to Rockall (57°N). Finally, when needed, loci were annotated by matching the SNP flanking regions against a non‐redundant database of GenBank (www.ncbi.nlm.nih.gov/genbank/) using the Basic Local Alignment Search Tool (Altschul et al. 1990).

## 3 Results

None of the loci deviated from neutral expectations in either BayeScan or Arlequin (Table S2), and consequently, all 61 loci were retained. The resolution power of the SNP array used was evidenced by the plateau of the genotype accumulation curve reached with 25% of the markers used, meaning that some 15 SNPs would be enough to differentiate unique individuals in a population (Figure S1).

The biplot resulting from Principal Components Analysis revealed a three‐stripped pattern lacking any underlying geographic basis (Figure S2a). The three resulting clusters contained different numbers of individuals and showed a pattern of allele frequency of 1–0.5–0, with the heterozygotes (CT) occupying the central position (Figure S2b). One of the homokaryotype groups was 5‐fold larger in number than the alternative one (457 vs. 86), whereas the number of heterozygote individuals was almost as large as the major homozygotes (*N* = 431). This pattern was driven by two strongly linked loci: Mdy033 and Mdy035 (Figure S3a,b), which could not be annotated to any predicted gene. Likewise, the major allele frequency per sample of these loci did not seem to adhere to any latitudinal pattern.

The dataset was LD‐pruned by removing locus Mdy035 thus leaving a dataset of 60 SNP loci for downstream analyses. Genetic diversity was similar across samples, with observed (*H*_o_) and unbiased expected heterozygosity (*uH*_e_) ranging between 0.330–0.374 and 0.330–0.365, respectively. No signs of inbreeding were detected and *F*_IS_ per sample ranged between −0.129 and 0.027 (Table 1). The PCA plot of this LD‐pruned dataset failed to show any clear geographic pattern (Figure S4). AMOVA conducted using a hierarchical approach revealed no differentiation due to fjords/offshore (*F*_CT_ = 0.001, *p* = 0.177), although locus Mdy002 and Mdy050 revealed significant *F*_CT_. The variation hosted among samples within groups was low yet significant (*F*_SC_ = 0.004, *p* < 0.0001) and primarily driven by the significant differentiation between Yrkefjorden and most of the samples within its group. Likewise, overall low but significant genetic differentiation was detected, with 99.5% of the variation hosted within samples (*F*_ST_ = 0.005, *p* < 0.0001). Most of the pairwise *F*_ST_ values ranged between 0 and 0.017 (Table 2), and the unevenness of the sampling sizes begs for caution when interpreting these values and its significance, e.g., the differentiation of the historic samples versus the small sample from the W_Fjords (*N* = 5). Although the overall differentiation was low, some samples showed significant structure. Thus, Iceland, Egga_South and Yrkefjord, which did not differ from each other, were different from most of the remaining samples. The significant differentiation detected between Faroe samples (*F*_ST_ = 0.002) is probably due to the statistical power of two samples of *N* ≈ 100. When considering the fjords, the only significant differentiation was registered between Yrkefjord versus Storfjord and Førdefjord. *F*_ST_ per locus was significant at 24 of the SNPs, with values ranging between 0.005 to 0.028.

**TABLE 2:** Genetic differentiation between geographically explicit samples at 60 LD‐pruned SNP loci: Pairwise *F*_ST_ values in the bottom diagonal and corresponding *p*‐values after 10,000 permutations in the top diagonal. Values in italics boldface font are significantly different from zero at *ɑ* = 0.05 whereas values in regular boldface font retained significance after FDR correction for multiple testing. Shaded cells in the first column identify samples with *N* ≤ 20.

	Nygrunnen	Iceland	Egga_S	Greenland	Storegg_93	Storegg_16_17	Faroe_15_16	Faroe_16_18	Rosemary B	Rockall Sl	Rockall	Rockall_76	Ryvingen	Skagerrak	Storfj	Fordefj	W_fjord	Yrkefj
**Nygrunnen**		0.754	0.504	0.343	0.139	0.139	0.088	0.080	0.838	***0.035***	0.153	0.584	0.867	0.426	0.189	0.189	0.407	0.826
**Iceland**	0.000		0.152	***0.048***	**0.000**	**0.000**	**0.000**	**0.000**	0.213	**0.000**	**0.000**	0.337	0.767	**0.000**	**0.000**	**0.002**	0.749	0.063
**Egga_S**	0.000	0.003		**0.007**	0.051	***0.033***	**0.001**	**0.001**	0.220	**0.010**	**0.001**	0.073	0.350	**0.002**	**0.002**	**0.000**	**0.014**	0.249
**Greenland**	0.002	0.004	0.011		**0.017**	0.669	0.377	0.745	0.315	0.310	0.260	0.996	0.497	0.324	0.986	0.487	***0.047***	**0.001**
**Storegg_93**	0.010	0.017	0.008	0.012		0.054	**0.009**	**0.012**	***0.036***	**0.004**	0.077	0.999	0.638	**0.002**	0.108	0.099	**0.006**	**0.000**
**Storegg_16_17**	0.006	0.007	0.006	0.000	0.006		0.183	0.379	0.165	0.501	0.475	1.000	0.692	0.656	0.885	0.332	0.120	**0.000**
**Faroe_15_16**	0.007	0.009	0.012	0.001	0.010	0.001		**0.029**	0.113	0.205	0.441	0.999	0.597	0.065	0.411	0.350	0.148	**0.000**
**Faroe_16_18**	0.007	0.012	0.011	0.000	0.009	0.000	0.002		0.115	0.613	0.283	1.000	0.346	0.059	0.738	0.658	**0.023**	**0.000**
**Rosemary B**	0.000	0.004	0.000	0.002	0.016	0.005	0.006	0.006		0.393	**0.041**	0.940	0.560	0.302	0.512	**0.016**	0.092	0.444
**Rockall Sl**	0.011	0.013	0.009	0.001	0.013	0.000	0.001	0.000	0.001		0.472	1.000	0.249	0.120	0.859	***0.049***	***0.041***	**0.000**
**Rockall**	0.006	0.010	0.012	0.001	0.006	0.000	0.000	0.001	0.010	0.000		1.000	0.632	**0.009**	0.838	0.482	0.053	**0.000**
**Rockall_76**	0.000	0.001	0.005	0.000	0.000	0.000	0.000	0.000	0.000	0.000	0.000		0.656	1.000	1.000	1.000	**0.006**	0.881
**Ryvingen**	0.000	0.000	0.000	0.000	0.000	0.000	0.000	0.003	0.000	0.007	0.000	0.000		0.786	0.319	0.404	0.678	0.898
**Skagerrak**	0.001	0.008	0.012	0.001	0.015	0.000	0.003	0.003	0.003	0.003	0.005	0.000	0.000		0.082	0.055	0.220	**0.004**
**Storfj**	0.005	0.010	0.013	0.000	0.005	0.000	0.000	0.000	0.000	0.000	0.000	0.000	0.004	0.004		0.553	***0.040***	**0.000**
**Fordefj**	0.005	0.007	0.015	0.000	0.006	0.001	0.001	0.000	0.013	0.004	0.000	0.000	0.001	0.004	0.000		0.061	**0.000**
**W_fjord**	0.003	0.000	0.027	0.022	0.048	0.014	0.012	0.023	0.017	0.020	0.020	0.046	0.000	0.010	0.021	0.018		0.527
**Yrkefj**	0.000	0.002	0.002	0.008	0.015	0.009	0.012	0.013	0.000	0.013	0.014	0.000	0.000	0.005	0.013	0.012	0.000	

**FIGURE 2:**
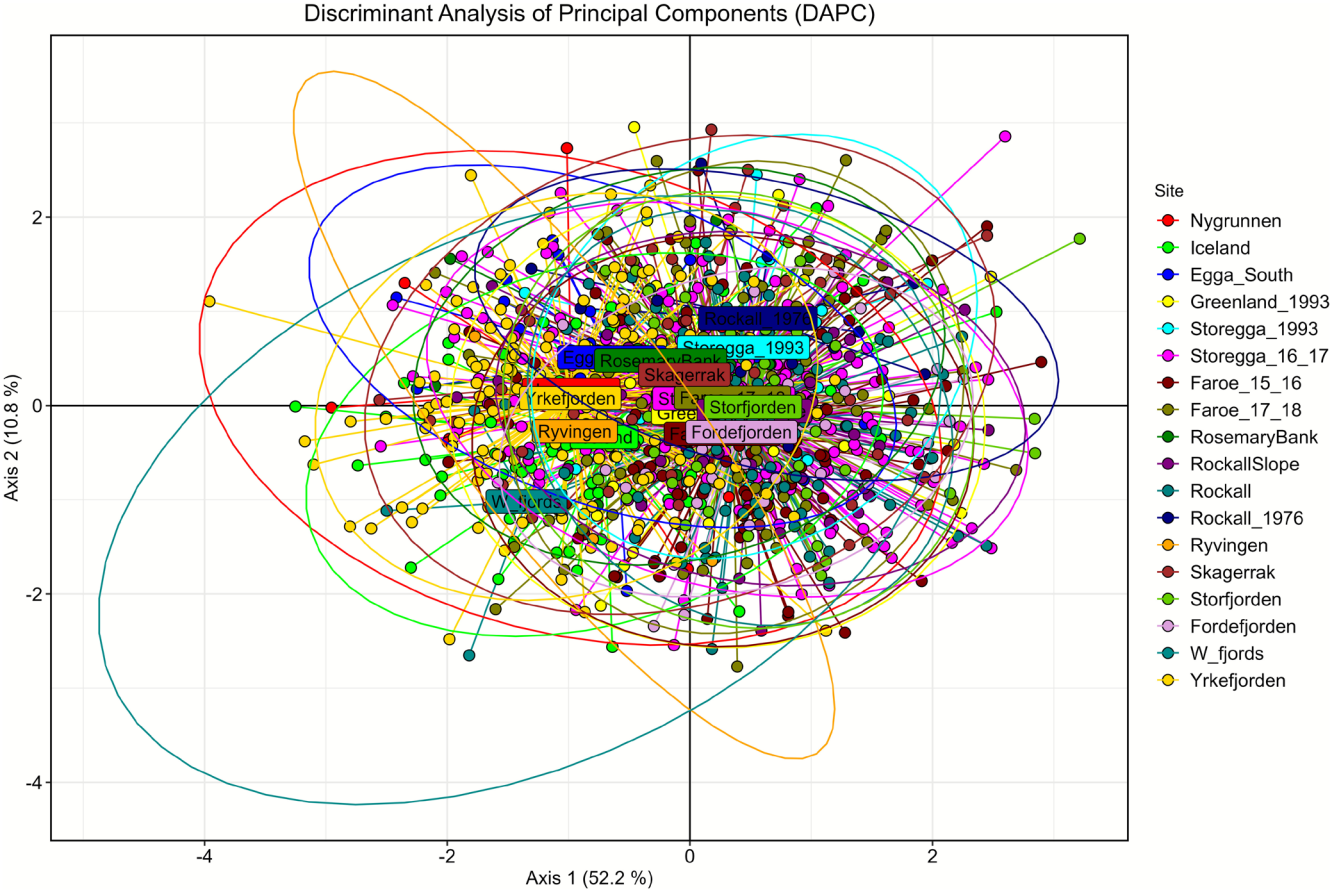
Genetic differentiation among blue ling samples assessed with 60 LD‐pruned SNP loci using Discriminant Analysis of Principal Components (DAPC) after retaining 15 principal components and three discriminant functions. Individuals from different sampling sites are represented by coloured dots, and name labels are placed on the centroid of the ellipse of each geographically explicit sample.

The DAPC was built after retaining 15 principal components and 3 discriminant functions. Despite the extensive overlapping, the first axis, which explained 52.2% of the variation, weakly separated the centres of inertia of the samples of Nygrunnen, Iceland, Yrkefjorden, Egga_South, Ryvingen and W_fjords (Figure 2). Axis 2 and 3, accounting for 10.8% and 9%, respectively, did not resolve further.

**FIGURE 3:**
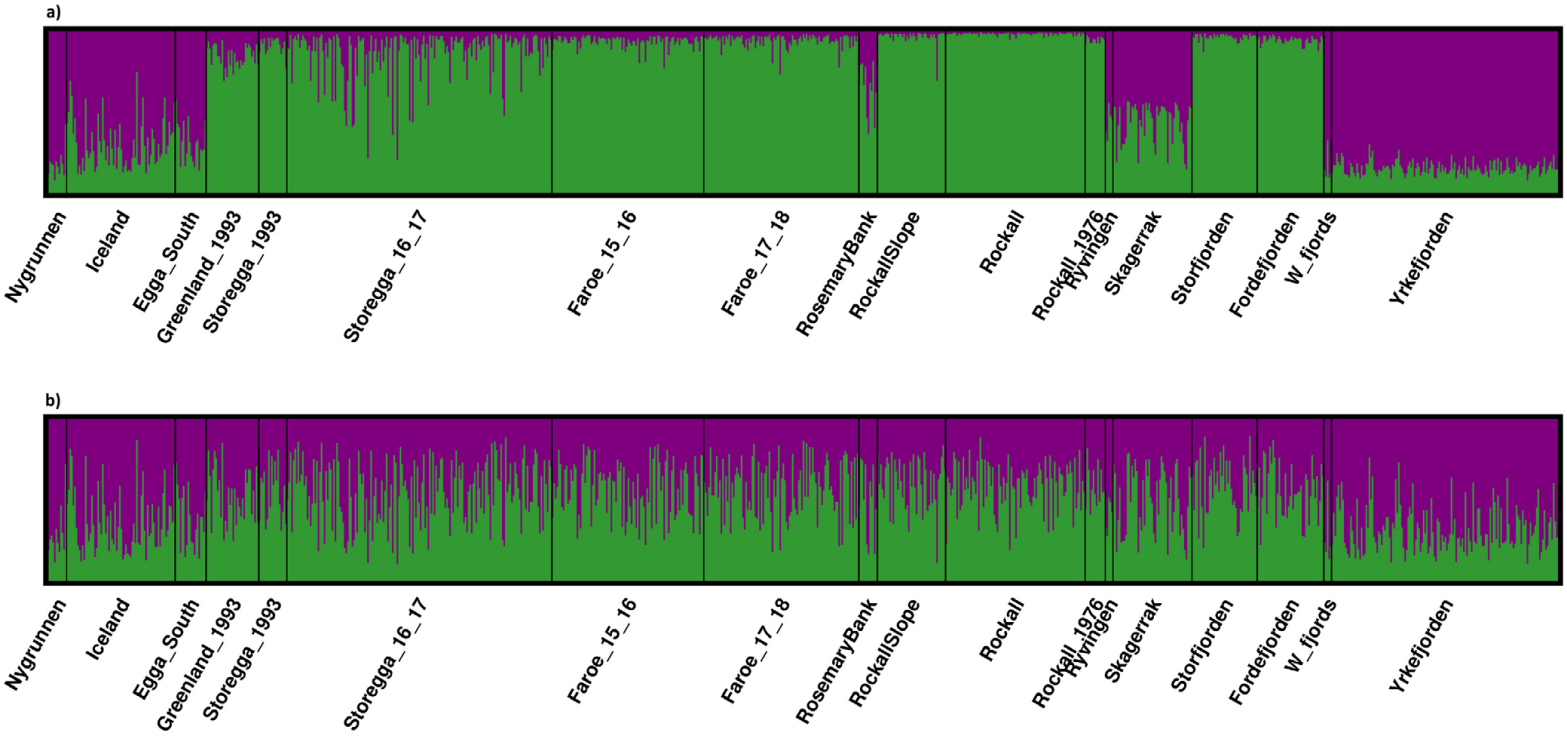
STRUCTURE a posteriori analysis: Barplot representing the proportion of individuals' ancestry to cluster at *K* = 2 as determined by both Evanno test and Puechmaille's statistics. STRUCTURE was conducted using the set of 60 LD‐pruned SNPs using geographic information to assist the clustering (a) and in a blind manner (b).

STRUCTURE conducted both with and without LOCPRIORS revealed that *K* = 2 was the most likely number of clusters according to both Puechmaille and Evanno's methods (Figure S5). The supervised analysis revealed that Nygrunnen, Iceland, Egga_South, Skagerrak, Rockall_1976, Ryvingen, W_fjords and Yrkefjorden differed from the remaining samples (Figure 3a). This pattern lost clarity when conducting STRUCTURE in an unsupervised manner without using geographic information to assist the clustering (Figure 3b). Rockall was sampled both in 1976 and 35 years later without any temporal change being detected through STRUCTURE barplots or pairwise *F*_ST_; however, it must be noted that in the historical sample, due to poor amplification, only 13 individuals remained. Likewise, no temporal differentiation was detected in Storegga in an interval of 24 years. No significant correlation was detected between genetic distance measured as *F*_ST_ and shortest water distance (*r*_xy_ = −0.120, *p* = 0.166) therefore revealing no Isolation by Distance using 60 LD‐pruned SNP loci.

Signs of latitudinal frequency clines were detected in 11 loci but only in eight; the difference between maximum and minimum frequency was ≥ 0.2. The permutation tests revealed that none of the clines except for locus Mdy085 was due to random processes (Table S3).

## 4 Discussion

The weak but significant genetic structure detected in this study using a panel of 60 LD‐pruned SNPs, as well as results from previously published literature (McGill et al. 2023) led to reject the hypothesis of panmixia in the Northeast Atlantic. Likewise, the hypothesis of a single ling stock in the Northeast Atlantic was challenged for the sister species, *Molva molva*, which revealed a longitudinal genetic break with Iceland and Rockall separated from the Faroese‐Norwegian coast cluster (Blanco González et al. 2015; McGill et al. 2023).

This study builds upon the existing knowledge on population connectivity in blue ling recently provided by McGill et al. (2023), who used a large SNP panel to genotype 190 individuals from the same geographic area as examined here. In contrast, in our study, close to 1000 individuals were genotyped with a small SNP panel formerly developed using ddRAD sequencing (Helle et al. 2020). Although aware of the limitations that such a reduced SNP representation imposes, the genotype accumulation curve suggested that this panel of markers carries enough power to discriminate between unique individuals. Likewise, former studies have shown not only that an extremely limited number of non‐diagnostic SNPs can provide extraordinary resolution in marine fish (Quintela et al. 2024; Seljestad et al. 2020), but also that patterns of differentiation initially detected with sparse molecular arrays received further confirmation from larger molecular sets including full genome sequencing (Andersson et al. 2017; Jansson et al. 2025; Pettersson et al. 2024).

Oceanographic barriers can modulate the movement of adult fish as well as the drifting of juveniles/larvae across major basins in the Northeast Atlantic and therefore shape genetic connectivity. The Wyville‐Thomson ridge (between the Hebrides and the Faroe Islands) offers favourable conditions for stratified water masses as the Norwegian Sea currents confluence with slower currents from the subtropical Atlantic Ocean (Hänninen 2020; Kurekin et al. 2020) and thus separates fish communities distributed along the Norwegian Sea and North Sea/Skagerrak from those to the west of the British Isles (Campbell et al. 2011). The barriers to gene flow that these hydrographic conditions create have been detected in deep‐sea fish species such as *Brosme brosme* (Knutsen et al. 2009) or *Coryphaenoides rupestris* (Longmore et al. 2011). Furthermore, the barrier that the Iceland‐Faroe Ridge shapes between the communities in the Norwegian Sea from those in the Icelandic basin (Gordon 1986) is reflected in the patterns of genetic differentiation found in blue ling between Iceland and Norwegian samples south to 62°N.

Eggs, larvae and small blue ling are found in all defined stock areas in the Northeast Atlantic (Bergstad 1991; ICES 2024). Fisheries assessments on blue ling from Iceland show that stock biomass has declined since 2012. At the same time, the biomass in the western slope of Scotland and Faroese waters has increased. If blue ling in these two areas were demographically connected, the decline in biomass seen at the Icelandic waters would be reflected in the Faroese waters, which is not the case (ICES 2024). The patterns of genetic differentiation detected here between Iceland and the samples from the Faroe Islands and west of Scotland further confirm the limited connectivity suggested by catch data.

The combined information provided by genetic markers together with knowledge about bathymetry and hydrographic conditions would support the differentiation of the two stocks in eastern and western areas of the North Atlantic. McGill et al. (2023) found significant differentiation in the NE Atlantic (56°–69°N) ranging from South Greenland to the Norwegian coast where Norway vs. Atlantic Ocean represented the major axis of differentiation. Atlantic samples in McGill et al. (2023) depict a homogenous cluster in contrast with ours, where significant structure was detected, e.g., between Iceland and most of the remaining ones. Some of our Norwegian samples overlap with McGill's; however, the low sample number (*N* = 5) of our W_fjord and Ryvingen samples does not allow us to make statistically sound comparisons. McGill et al. (2023) reported very low but significant differentiation across Norwegian samples, and here, differentiation was detected between Yrkefjorden and Storfjorden/Førdefjorden (*F*_ST_ ≈ 0.012). These samples come from sheltered fjord systems with shallow sills or shoals (bedrock) at their mouth created during the last ice age (Murton et al. 2006). These shallow areas reduce the inflow and outflow of water and most likely reduce the migration of a deep‐water fish, like blue ling, in and out of the fjords. Hence, it would likely result in genetically isolated populations in these fjord systems, in agreement with the observed in mesopelagic species (Quintela et al. 2024, 2025).

The fjord vs. offshore differentiation is mainly driven by Yrkefjorden, although with exceptions as no differentiation was found when comparing with Iceland (Nygrunnen, Egga_South and Rosemary Bank need to be taken with caution due to low *N*). Thus, the strong fjord vs. offshore patterns formerly described in gadoids (Hill 2021; Johansen et al. 2020; Knutsen et al. 2009; Myksvoll et al. 2022; Saha et al. 2015; Sodeland et al. 2016; Westgaard et al. 2017) or small pelagic and mesopelagic fish (Pettersson et al. 2024; Quintela et al. 2024, 2020, 2025) need to be further explored for blue ling with a more comprehensive markers array as no clear fjord‐offshore differentiation was detected thus far.

Finally, our results might suggest the existence of a putative structural variant as revealed by the triple striation displayed in the PCA biplot produced before LD‐pruning. The utility of PCA for the detection and characterisation of chromosome inversions was initiated by Ma and Amos (2012). PCA striations detected in polar cod using modest SNP datasets and suggestive of eventual chromosome inversions (Quintela et al. 2021) were confirmed by full genome sequencing (Bringloe et al. 2024; Maes et al. 2025). The growing body of literature dealing with chromosome inversions in Atlantic cod reported that they underlie four supergenes allegedly linked to migratory lifestyle and adaptations to, e.g., salinity (Matschiner et al. 2022). Besides, inversions in chromosomes 2, 7 and 12 have been identified in coastal vs. offshore samples of Atlantic cod (Johansen et al. 2020; Sodeland et al. 2016), whereas chromosome 2 has been shown to be highly divergent between spring and winter spawners within the Gulf of Maine (Barney et al. 2017). The taxonomic relatedness between Atlantic cod and blue ling, both belonging to the same order, makes it not unlikely that chromosome inversions happen in the latter although genomic tools are needed to verify this extent and, if so, to identify the environmental factor driving them. Thus, if inversions were confirmed in the blue ling genome, they should be taken into consideration when attempting to produce loci arrays for the delineation of management units as suggested by Pita et al. (2022) as they represent a source of evolutionary novelty that can be crucial to ensure sustainability.

## 5 Management Implications

The differentiation formerly described between blue ling from eastern and western areas of the North Atlantic was strengthened by our results. However, we were unable to identify neat differentiation between fjord‐offshore populations for blue ling in Norwegian waters. There are some indications of isolated populations in sheltered fjord systems but the interactions between fjord/coastal and offshore populations need to be further investigated with more powerful molecular tools. Information about such interactions is important for management of blue ling as smaller, isolated populations would be more vulnerable than larger offshore populations with more resilience to anthropogenic changes such as climate change, habitat alteration or human exploitation.

## 6 Conclusions

All loci used in the present study conformed to neutral expectations and, although no clear geographic structure emerged after LD‐pruning, low but significant differentiation was detected, largely driven by the distinctiveness of Yrkefjorden and, to a lesser extent, Iceland and Egga_South. Clustering analyses revealed only weak patterns, and no isolation‐by‐distance was detected. A limited number of loci showed significant latitudinal clines, with only one (Mdy085) unlikely to result from random processes.

While results using 60 SNPs detected some genetic differentiation in this study, they did not reveal the same level of structure observed in other studies (McGill et al. 2023), indicating that additional variation likely exists. Although results suggest high connectivity overall, they also highlight the need for broader fjord sampling, as shown in this study, and the use of larger SNP panels to achieve finer resolution.

## Author Contributions

**María Quintela:** formal analysis (lead), methodology (equal), visualization (lead), writing – original draft (equal), writing – review and editing (equal). **Hege Øverbø Hansen:** data curation (lead), writing – original draft (equal), writing – review and editing (supporting). **Laila Unneland:** investigation (equal), methodology (lead), validation (lead). **Ilaria Coscia:** data curation (supporting), writing – original draft (supporting), writing – review and editing (equal). **Torild Johansen:** data curation (supporting), methodology (supporting), writing – review and editing (supporting). **Lise Helen Ofstad:** data curation (equal), writing – original draft (supporting), writing – review and editing (equal). **Kristin Helle:** conceptualization (lead), data curation (equal), funding acquisition (lead), project administration (lead), writing – original draft (supporting), writing – review and editing (supporting).

## Funding

The study was funded by the Norwegian Directorate of Fisheries and the Norwegian Fishermen's Association. The authors contributed to the text, agreed with its content and approved it for submission.

## Ethics Statement

The fish used in this study were caught during monitoring surveys performed by the Norwegian Institute of Marine Research in combination with the Norwegian Reference Fleet using standard sampling routines to meet scientific and ethical concerns. Blue ling is a commercial species that was collected in scientific surveys where all research met the ethical guidelines of the study countries.

## Conflicts of Interest

The authors declare no conflicts of interest.

## Supporting information


**Data S1:** ece372801‐sup‐0001‐supinfo.docx.

## Data Availability

Genotype raw data used in this study can be publicly accessed from the electronic archive of the Institute of Marine Research at https://hdl.handle.net/11250/3211560.

## References

[ece372801-bib-0001] Allendorf, F. W. , P. R. England , G. Luikart , P. A. Ritchie , and N. Ryman . 2008. “Genetic Effects of Harvest on Wild Animal Populations.” Trends in Ecology & Evolution 23, no. 6: 327–337. 10.1016/j.tree.2008.02.008. 18439706

[ece372801-bib-0002] Altschul, S. F. , W. Gish , W. Miller , E. W. Myers , and D. J. Lipman . 1990. “Basic Local Alignment Search Tool.” Journal of Molecular Biology 215, no. 3: 403–410. 10.1016/S0022-2836(05)80360-2. 2231712

[ece372801-bib-0003] Andersson, A. , E. Jansson , L. Wennerström , et al. 2017. “Complex Genetic Diversity Patterns of Cryptic, Sympatric Brown Trout ( *Salmo trutta* ) Populations in Tiny Mountain Lakes.” Conservation Genetics 18, no. 5: 1213–1227. 10.1007/s10592-017-0972-4.

[ece372801-bib-0004] Andersson, L. , D. Bekkevold , F. Berg , et al. 2024. “How Fish Population Genomics Can Promote Sustainable Fisheries: A Road Map.” Annual Review of Animal Biosciences 12: 1–20. 10.1146/annurev-animal-021122-102933. 37906837

[ece372801-bib-0005] Barney, B. T. , C. Munkholm , D. R. Walt , and S. R. Palumbi . 2017. “Highly Localized Divergence Within Supergenes in Atlantic Cod (*Gadus morhua*) Within the Gulf of Maine.” BMC Genomics 18, no. 1: 271. 10.1186/s12864-017-3660-3. 28359300 PMC5374575

[ece372801-bib-0006] Benjamini, Y. , and Y. Hochberg . 1995. “Controlling the False Discovery Rate: A Practical and Powerful Approach to Multiple Testing.” Journal of the Royal Statistical Society. Series B, Statistical Methodology 57, no. 1: 289–300. 10.1111/j.2517-6161.1995.tb02031.x.

[ece372801-bib-0007] Bergstad, O. A. 1991. “Distribution and Trophic Ecology of Some Gadoid Fish of the Norwegian Deep.” Sarsia 75, no. 4: 269–313. 10.1080/00364827.1991.10413455.

[ece372801-bib-0008] Besnier, F. , and K. A. Glover . 2013. “ParallelStructure: A R Package to Distribute Parallel Runs of the Population Genetics Program STRUCTURE on Multi‐Core Computers.” PLoS One 8, no. 7: e70651. 10.1371/journal.pone.0070651. 23923012 PMC3726640

[ece372801-bib-0009] Blanco González, E. , H. Knutsen , P. E. Jorde , K. A. Glover , and O. A. Bergstad . 2015. “Genetic Analyses of Ling ( *Molva molva* ) in the Northeast Atlantic Reveal Patterns Relevant to Stock Assessments and Management Advice.” ICES Journal of Marine Science 72, no. 2: 635–641. 10.1093/icesjms/fsu135.

[ece372801-bib-0010] Bringloe, T. T. , A. Bourret , D. Cote , et al. 2024. “Genomic Architecture and Population Structure of *Boreogadus saida* From Canadian Waters.” Scientific Reports 14, no. 1: 19331. 10.1038/s41598-024-69782-w. 39164428 PMC11336163

[ece372801-bib-0011] Cadrin, S. X. , L. A. Kerr , and S. Mariani . 2014. Stock Identification Methods: Applications in Fishery Science. Second edition ed. Academic Press.

[ece372801-bib-0012] Campbell, N. , F. Neat , F. Burns , and P. Kunzlik . 2011. “Species Richness, Taxonomic Diversity, and Taxonomic Distinctness of the Deep‐Water Demersal Fish Community on the Northeast Atlantic Continental Slope (ICES Subdivision VIa).” ICES Journal of Marine Science 68, no. 2: 365–376. 10.1093/icesjms/fsq070.

[ece372801-bib-0013] Cohen, D. M. , T. Inada , T. Iwamoto , and N. Scialabba . 1990. FAO Species Catalogue. Vol. 10. Gadiform Fishes of the World (Order Gadiformes). An Annotated and Illustrated Catalogue of Cods, Hakes, Grenadiers and Other Gadiform Fishes Known to Date. Vol. 10. FAO Fisheries Synopsis.

[ece372801-bib-0086] Derryberry, E. P. , G. E. Derryberry , J. M. Maley , and R. T. Brumfield . 2014. “HZAR: hybrid zone analysis using an R software package.” Molecular Ecology Resources 14, no. 3: 652–663. 10.1111/1755-0998.12209. 24373504

[ece372801-bib-0014] Dray, S. , and A.‐B. Dufour . 2007. “The ade4 Package: Implementing the Duality Diagram for Ecologists.” Journal of Statistical Software 22, no. 4: 1–20. 10.18637/jss.v022.i04.

[ece372801-bib-0015] Ehrich, S. 1982. “Fishery Biological Investigations on Blue Ling Stocks in the Area North of Rockall Deep.” Informationen Fischwirtschaft 29: 53–55.

[ece372801-bib-0016] Engås, A. 1983. “Betydning av Ulike Redskapsfaktorer i Garnfisket Etter Blålange, *M. dypterygia*.” Hovedfag i Fiskeribiolgi, Institutt for Fiskeribiologi, Universitetet i Bergen.

[ece372801-bib-0017] Evanno, G. , S. Regnaut , and J. Goudet . 2005. “Detecting the Number of Clusters of Individuals Using the Software STRUCTURE: A Simulation Study.” Molecular Ecology 14, no. 8: 2611–2620. 10.1111/j.1365-294X.2005.02553.x. 15969739

[ece372801-bib-0018] Excoffier, L. , G. Laval , and S. Schneider . 2005. “Arlequin ver. 3.0: An Integrated Software Package for Population Genetics Data Analysis.” Evolutionary Bioinformatics Online 1: 47–50. 10.1177/117693430500100003. PMC2658868 19325852

[ece372801-bib-0019] Foll, M. , and O. Gaggiotti . 2006. “Identifying the Environmental Factors That Determine the Genetic Structure of Populations.” Genetics 174, no. 2: 875–891. 10.1534/genetics.106.059451. 16951078 PMC1602080

[ece372801-bib-0020] Foll, M. , and O. Gaggiotti . 2008. “A Genome‐Scan Method to Identify Selected Loci Appropriate for Both Dominant and Codominant Markers: A Bayesian Perspective.” Genetics 180, no. 2: 977–993. 10.1534/genetics.108.092221. 18780740 PMC2567396

[ece372801-bib-0021] Froese, R. , and D. Pauly . 2023. “FishBase.” World Wide Web Electronic Publication. www.fishbase.org.

[ece372801-bib-0022] Funk, W. C. , J. K. McKay , P. A. Hohenlohe , and F. W. Allendorf . 2012. “Harnessing Genomics for Delineating Conservation Units.” Trends in Ecology & Evolution 27, no. 9: 489–496. 10.1016/j.tree.2012.05.012. 22727017 PMC4185076

[ece372801-bib-0023] Gabriel, S. , L. Ziaugra , and D. Tabbaa . 2009. “SNP Genotyping Using the Sequenom MassARRAY iPLEX Platform.” Current Protocols in Human Genetics 60, no. 1: 60. 10.1002/0471142905.hg0212s60. 19170031

[ece372801-bib-0024] Gordon, J. , and J. E. Hunter . 1994. “Study of Deep‐Water Fish Stocks to the West of Scotland.” SAMS Internal Reports; No. 194. Scottish Association for Marine Science.

[ece372801-bib-0025] Gordon, J. D. M. 1986. “The Fish Populations of the Rockall Trough.” Proceedings of the Royal Society of Edinburgh. Section B. Biological Sciences 88: 191–204. 10.1017/S0269727000004553.

[ece372801-bib-0026] Hänninen, K. I. 2020. “Driving Forces and Flow Mechanisms of the Atlantic Ocean Currents.” Environment and Ecology Research 8: 1–28. 10.13189/eer.2020.080101.

[ece372801-bib-0027] Helle, K. , M. Quintela , J. B. Taggart , et al. 2020. “Development of SNP for the Deep‐Sea Fish Blue Ling, *Molva dypterygia* (Pennant, 1784) From ddRAD Sequencing Data.” Conservation Genetics Resources 12, no. 2: 231–237. 10.1007/s12686-019-01107-w.

[ece372801-bib-0028] Hill, G. 2021. “Transcriptomic Basis for Differentiation of Fjord and Offshore *Boreogadus saida* (Polar Cod) Populations.” In Department of Arctic and Marine Biology, Faculty of Biosciences, Fisheries and Economics (Vol. MASTER'S thesis in Biology), 61. Artic University of Norway.

[ece372801-bib-0029] Hislop, J. , O. A. Bergstad , T. Jakobsen , et al. 2015. “Blue Ling – *Molva dypterygia* s.1.” In Fish Atlas of the Celtic Sea, North Sea and Baltic Sea, edited by H. J. L. Heessen , N. Daan , and J. R. Ellis , 231–232. Wageningen Academic Publishers and KNNV Publishing.

[ece372801-bib-0030] Hubisz, M. , D. Falush , M. Stephens , and J. Pritchard . 2009. “Inferring Weak Population Structure With the Assistance of Sample Group Information.” Molecular Ecology Resources 9, no. 5: 1322–1332. 10.1111/j.1755-0998.2009.02591.x. 21564903 PMC3518025

[ece372801-bib-0031] ICES . 2018. “Blue Ling (*Molva dypterygia*) in Subareas 6–7 and Division 5.B (Celtic Seas, English Channel, and Faroes grounds).” Report of the ICES Advisory Committee, 2018. ICES Advice 2018. bli‐5b67. 10.17895/ices.pub.4400.

[ece372801-bib-0032] ICES . 2024. “Report of the Working Group on the Biology and Assessment of Deep‐Sea Fisheries Resources (WGDEEP).” ICES Scientific Reports 6: 1156. 10.17895/ices.pub.25964749.

[ece372801-bib-0033] Jakobsson, M. , and N. A. Rosenberg . 2007. “CLUMPP: A Cluster Matching and Permutation Program for Dealing With Label Switching and Multimodality in Analysis of Population Structure.” Bioinformatics 23, no. 14: 1801–1806. 10.1093/bioinformatics/btm233. 17485429

[ece372801-bib-0034] Jansson, E. , F. Ayllon , C. J. Rubin , et al. 2025. “Genomic Landscape of Divergence in Ballan Wrasse (*Labrus bergylta*).” Molecular Ecology 34: e17732. 10.1111/mec.17732. 40095420 PMC12573741

[ece372801-bib-0035] Johansen, T. , F. Besnier , M. Quintela , et al. 2020. “Genomic Analysis Reveals Neutral and Adaptive Patterns That Challenge the Current Management Regime for East Atlantic Cod *Gadus morhua* L.” Evolutionary Applications 13, no. 10: 2673–2688. 10.1111/eva.13070. 33294016 PMC7691467

[ece372801-bib-0036] Jombart, T. 2008. “ *Adegenet*: A R Package for the Multivariate Analysis of Genetic Markers.” Bioinformatics 24: 1403–1405. 10.1093/bioinformatics/btn129. 18397895

[ece372801-bib-0037] Jombart, T. , and C. Collins . 2015. “A Tutorial for Discriminant Analysis of Principal Components (DAPC) Using Adegenet 2.0.0.” https://adegenet.r‐forge.r‐project.org/files/tutorial‐dapc.pdf.

[ece372801-bib-0038] Jombart, T. , S. Devillard , and F. Balloux . 2010. “Discriminant Analysis of Principal Components: A New Method for the Analysis of Genetically Structured Populations.” BMC Genetics 11, no. 1: 94. 10.1186/1471-2156-11-94. 20950446 PMC2973851

[ece372801-bib-0039] Jorde, P. E. , M. B. O. Huserbråten , B. B. Seliussen , et al. 2021. “The Making of a Genetic Cline: Introgression of Oceanic Genes Into Coastal Cod Populations in the Northeast Atlantic.” Canadian Journal of Fisheries and Aquatic Sciences 78, no. 7: 958–968. 10.1139/cjfas-2020-0380.

[ece372801-bib-0040] Kålås, J. A. , Å. Viken , and T. Bakken . 2006. Norsk Rødliste 2006–2006 Norwegian Red List. Artsdatabanken.

[ece372801-bib-0041] Kamvar, Z. N. , J. F. Tabima , and N. J. Grünwald . 2014. “Poppr: An R Package for Genetic Analysis of Populations With Clonal, Partially Clonal, and/or Sexual Reproduction.” PeerJ 2: e281. 10.7717/peerj.281. 24688859 PMC3961149

[ece372801-bib-0042] Kerr, L. A. , N. T. Hintzen , S. X. Cadrin , et al. 2017. “Lessons Learned From Practical Approaches to Reconcile Mismatches Between Biological Population Structure and Stock Units of Marine Fish.” ICES Journal of Marine Science 74, no. 6: 1708–1722. 10.1093/icesjms/fsw188.

[ece372801-bib-0043] Knutsen, H. , P. E. Jorde , H. Sannæs , et al. 2009. “Bathymetric Barriers Promoting Genetic Structure in the Deepwater Demersal Fish Tusk (*Brosme brosme*).” Molecular Ecology 18, no. 15: 3151–3162. 10.1111/j.1365-294X.2009.04253.x. 19549108

[ece372801-bib-0044] Kurekin, A. A. , P. E. Land , and P. I. Miller . 2020. “Internal Waves at the UK Continental Shelf: Automatic Mapping Using the ENVISAT ASAR Sensor.” Remote Sensing 12, no. 15: 2476. 10.3390/rs12152476.

[ece372801-bib-0045] Large, P. A. , G. Diez , J. Drewery , et al. 2010. “Spatial and Temporal Distribution of Spawning Aggregations of Blue Ling ( *Molva dypterygia* ) West and Northwest of the British Isles.” ICES Journal of Marine Science 67, no. 3: 494–501. 10.1093/icesjms/fsp264.

[ece372801-bib-0046] Li, Y.‐L. , and J.‐X. Liu . 2018. “StructureSelector: A Web‐Based Software to Select and Visualize the Optimal Number of Clusters Using Multiple Methods.” Molecular Ecology Resources 18, no. 1: 176–177. 10.1111/1755-0998.12719. 28921901

[ece372801-bib-0047] Longmore, C. , C. N. Trueman , F. Neat , E. J. O'Gorman , J. A. Milton , and S. Mariani . 2011. “Otolith Geochemistry Indicates Life‐Long Spatial Population Structuring in a Deep‐Sea Fish, *Coryphaenoides rupestris* .” Marine Ecology Progress Series 435: 209–224. 10.3354/meps09197.

[ece372801-bib-0048] Ma, J. , and C. I. Amos . 2012. “Investigation of Inversion Polymorphisms in the Human Genome Using Principal Components Analysis.” PLoS One 7, no. 7: e40224. 10.1371/journal.pone.0040224. 22808122 PMC3392271

[ece372801-bib-0049] Maes, S. M. , M. L. Verheye , C. Bouchard , et al. 2025. “Reduced‐Representation Sequencing Detects Trans‐Arctic Connectivity and Local Adaptation in Polar Cod ( *Boreogadus saida* ).” Molecular Ecology 34, no. 7: e17706. 10.1111/mec.17706. 40040553 PMC11934089

[ece372801-bib-0050] Magnússon, J. V. , O. A. Bergstad , N.‐R. Hareide , J. Magnússon , and J. Reinert . 1997. Ling, Blue Ling and Tusk of the Northeast Atlantic. Nordic Council of Ministers.

[ece372801-bib-0051] Mantel, N. 1967. “The Detection of Disease of Clustering and a Generalized Regression Approach.” Cancer Research 27, no. 2: 209–220. 6018555

[ece372801-bib-0052] Matschiner, M. , J. M. I. Barth , O. K. Tørresen , et al. 2022. “Origin and Fate of Supergenes in Atlantic Cod.” Nature Ecology & Evolution 6: 469–484. 10.1038/s41559-022-01661-x. 35177802 PMC8986531

[ece372801-bib-0053] McGill, L. , A. D. McDevitt , B. Hellemans , et al. 2023. “Population Structure and Connectivity in the Genus *Molva* in the Northeast Atlantic.” ICES Journal of Marine Science 80, no. 4: 1079–1086. 10.1093/icesjms/fsad040.

[ece372801-bib-0054] Miller, J. M. , C. I. Cullingham , and R. M. Peery . 2020. “The Influence of a Priori Grouping on Inference of Genetic Clusters: Simulation Study and Literature Review of the DAPC Method.” Heredity 125, no. 5: 269–280. 10.1038/s41437-020-0348-2. 32753664 PMC7553915

[ece372801-bib-0055] Murton, J. B. , R. Peterson , and J.‐C. Ozouf . 2006. “Bedrock Fracture by Ice Segregation in Cold Regions.” Science 314, no. 5802: 1127–1129. 10.1126/science.1132127. 17110573

[ece372801-bib-0056] Myksvoll, M. S. , J. Devine , M. Quintela , et al. 2022. “Linking Dispersal Connectivity to Population Structure and Management Boundaries for Saithe, *Pollachius virens* (Linnaeus, 1758) in the Northeast Atlantic.” Marine Ecology Progress Series 680: 177–191. 10.3354/meps13862.

[ece372801-bib-0057] Pante, E. , and B. Simon‐Bouhet . 2013. “Marmap: A Package for Importing, Plotting and Analyzing Bathymetric and Topographic Data in R.” PLoS One 8, no. 9: e73051. 10.1371/journal.pone.0073051. 24019892 PMC3760912

[ece372801-bib-0058] Peakall, R. , and P. E. Smouse . 2006. “GenAlEx 6: Genetic Analysis in Excel. Population Genetic Software for Teaching and Research.” Molecular Ecology Notes 6, no. 1: 288–295. 10.1111/j.1471-8286.2005.01155.x. PMC3463245 22820204

[ece372801-bib-0059] Pereira, P. , J. Teixeira , and G. Velo‐Antón . 2018. “Allele Surfing Shaped the Genetic Structure of the European Pond Turtle via Colonization and Population Expansion Across the Iberian Peninsula From Africa.” Journal of Biogeography 45, no. 9: 2202–2215. 10.1111/jbi.13412.

[ece372801-bib-0060] Pettersson, M. E. , M. Quintela , F. Besnier , et al. 2024. “Limited Parallelism in Genetic Adaptation to Brackish Water Bodies in European Sprat and Atlantic Herring.” Genome Biology and Evolution 16, no. 7: evae133. 10.1093/gbe/evae133. 38918882 PMC11226789

[ece372801-bib-0061] Pinsky, M. L. , and S. R. Palumbi . 2014. “Meta‐Analysis Reveals Lower Genetic Diversity in Overfished Populations.” Molecular Ecology 23, no. 1: 29–39. 10.1111/mec.12509. 24372754

[ece372801-bib-0062] Pita, A. , M. Fernández‐Míguez , and P. Presa . 2022. “EST‐Microsatellite Types and Structural Scenarios in European Hake Fisheries.” Animals 12, no. 11: 1462. 10.3390/ani12111462. 35681926 PMC9179439

[ece372801-bib-0063] Priede, I. G. 2019. “Deep‐Sea Fishes Literature Review, JNCC Report No. 619.” JNCC: Peterborough. ISSN 0963–8091.

[ece372801-bib-0064] Pritchard, J. K. , M. Stephens , and P. Donnelly . 2000. “Inference of Population Structure Using Multilocus Genotype Data.” Genetics 155, no. 2: 945–959. 10.1093/genetics/155.2.945. 10835412 PMC1461096

[ece372801-bib-0065] Puechmaille, S. J. 2016. “The Program Structure Does Not Reliably Recover the Correct Population Structure When Sampling Is Uneven: Subsampling and New Estimators Alleviate the Problem.” Molecular Ecology Resources 16, no. 3: 608–627. 10.1111/1755-0998.12512. 26856252

[ece372801-bib-0066] Quintela, M. , S. Bhat , K. Præbel , et al. 2021. “Distinct Genetic Clustering in the Weakly Differentiated Polar Cod, *Boreogadus saida* Lepechin, 1774 From East Siberian Sea to Svalbard.” Polar Biology 44: 1711–1724. 10.1007/s00300-021-02911-7.

[ece372801-bib-0067] Quintela, M. , E. García‐Seoane , G. Dahle , et al. 2024. “Genetics in the Ocean's Twilight Zone: Population Structure of the Glacier Lanternfish Across Its Distribution Range.” Evolutionary Applications 17: e70032. 10.1111/eva.70032. 39513049 PMC11540841

[ece372801-bib-0068] Quintela, M. , C. Kvamme , D. Bekkevold , et al. 2020. “Genetic Analysis Redraws the Management Boundaries for the European Sprat.” Evolutionary Applications 13: 1906–1922. 10.1111/eva.12942. 32908594 PMC7463317

[ece372801-bib-0069] Quintela, M. , A. Mateos‐Rivera , R. Lille‐Langøy , et al. 2025. “Genetics in the Ocean's Twilight Zone: Population Structure of the Silvery Lightfish Across Its Distribution Range.” *Evolutionary Applications*. 10.1111/eva.70188. PMC13093611 42015953

[ece372801-bib-0078] R Core Team . 2025. “R: A Language and Environment for Statistical Computing.” R Foundation for Statistical Computing: Vienna, Austria. https://www.R‐project.org/.

[ece372801-bib-0070] Reiss, H. , G. Hoarau , M. Dickey‐Collas , and W. J. Wolff . 2009. “Genetic Population Structure of Marine Fish: Mismatch Between Biological and Fisheries Management Units.” Fish and Fisheries 10, no. 4: 361–395. 10.1111/j.1467-2979.2008.00324.x.

[ece372801-bib-0071] Rosenberg, M. S. , and C. D. Anderson . 2011. “PASSaGE: Pattern Analysis, Spatial Statistics and Geographic Exegesis. Version 2.” Methods in Ecology and Evolution 2, no. 3: 229–232. 10.1111/j.2041-210X.2010.00081.x.

[ece372801-bib-0072] Rousset, F. 1997. “Genetic Differentiation and Estimation of Gene Flow From F‐Statistics Under Isolation by Distance.” Genetics 145, no. 4: 1219–1228. 10.1093/genetics/145.4.1219. 9093870 PMC1207888

[ece372801-bib-0073] Rousset, F. 2008. “GENEPOP'007: A Complete Re‐Implementation of the Genepop Software for Windows and Linux.” Molecular Ecology Resources 8, no. 1: 103–106. 10.1111/j.1471-8286.2007.01931.x. 21585727

[ece372801-bib-0074] Saha, A. , L. Hauser , M. Kent , et al. 2015. “Seascape Genetics of Saithe ( *Pollachius virens* ) Across the North Atlantic Using Single Nucleotide Polymorphisms.” ICES Journal of Marine Science 72, no. 9: 2732–2741. 10.1093/icesjms/fsv139.

[ece372801-bib-0075] Seljestad, G. W. , M. Quintela , E. Faust , et al. 2020. ““A Cleaner Break”: Genetic Divergence Between Geographic Groups and Sympatric Phenotypes Revealed in Ballan Wrasse ( *Labrus bergylta* ).” Ecology and Evolution 10, no. 12: 6120–6135. 10.1002/ece3.6404. 32607218 PMC7319121

[ece372801-bib-0076] Slatkin, M. 1993. “Isolation by Distance in Equilibrium and Non‐Equilibrium Populations.” Evolution 47, no. 1: 264–279. 10.1111/j.1558-5646.1993.tb01215.x. 28568097

[ece372801-bib-0077] Sodeland, M. , P. E. Jorde , S. Lien , et al. 2016. ““Islands of Divergence” in the Atlantic Cod Genome Represent Polymorphic Chromosomal Rearrangements.” Genome Biology and Evolution 8, no. 4: 1012–1022. 10.1093/gbe/evw057. 26983822 PMC4860689

[ece372801-bib-0079] Thomas, R. 1987. “Biological Investigations on the Blue Ling, *Molva dypterygia dypterygia* (Pennan 1784 After O. F. Müller 1776), in the Areas of The Faroe Islands and to the West of the Shetland Islands.” Archiv für Fischereiwissenschaft 38: 9–34.

[ece372801-bib-0080] Waples, R. S. , A. E. Punt , and J. M. Cope . 2008. “Integrating Genetic Data Into Management of Marine Resources: How Can We Do It Better?” Fish and Fisheries 9, no. 4: 423–449. 10.1111/j.1467-2979.2008.00303.x.

[ece372801-bib-0081] Weir, B. S. , and C. Cockerham . 1984. “Estimating F‐Statistics for the Analysis of Population Structure.” Evolution 38, no. 6: 1358–1370. 10.2307/2408641. 28563791

[ece372801-bib-0082] Westgaard, J.‐I. , A. Staby , J. Aanestad Godiksen , et al. 2017. “Large and Fine Scale Population Structure in European Hake ( *Merluccius merluccius* ) in the Northeast Atlantic.” ICES Journal of Marine Science 74, no. 5: 1300–1310. 10.1093/icesjms/fsw249.

[ece372801-bib-0083] Wheeler, A. 1969. The Fishes of the British Isles and North‐West Europe. Michigan State University Press.

[ece372801-bib-0084] Wickham, H. 2016. ggplot2: Elegant Graphics for Data Analysis. Springer‐Verlag New York.

[ece372801-bib-0085] Wright, S. 1943. “Isolation by Distance.” Genetics 28, no. 2: 114–138. 10.1093/genetics/28.2.114. 17247074 PMC1209196

