# Supplementary material for: Genetic Structure of Blue Ling, Molva dypterygia, in the North Atlantic
Source: Ecol Evol. 2026 Mar 27;16(4):e72801. doi: 10.1002/ece3.72801 (PMC13107286; doi:10.1002/ece3.72801)
Supplement: Supplementary file 1 — Data S1: ece372801‐sup‐0001‐supinfo.docx. [file ECE3-16-e72801-s001.docx]

**SUPPLEMENT**

**Genetic structure of blue ling, *Molva dypterygia* in the North Atlantic**

**FIGURES**


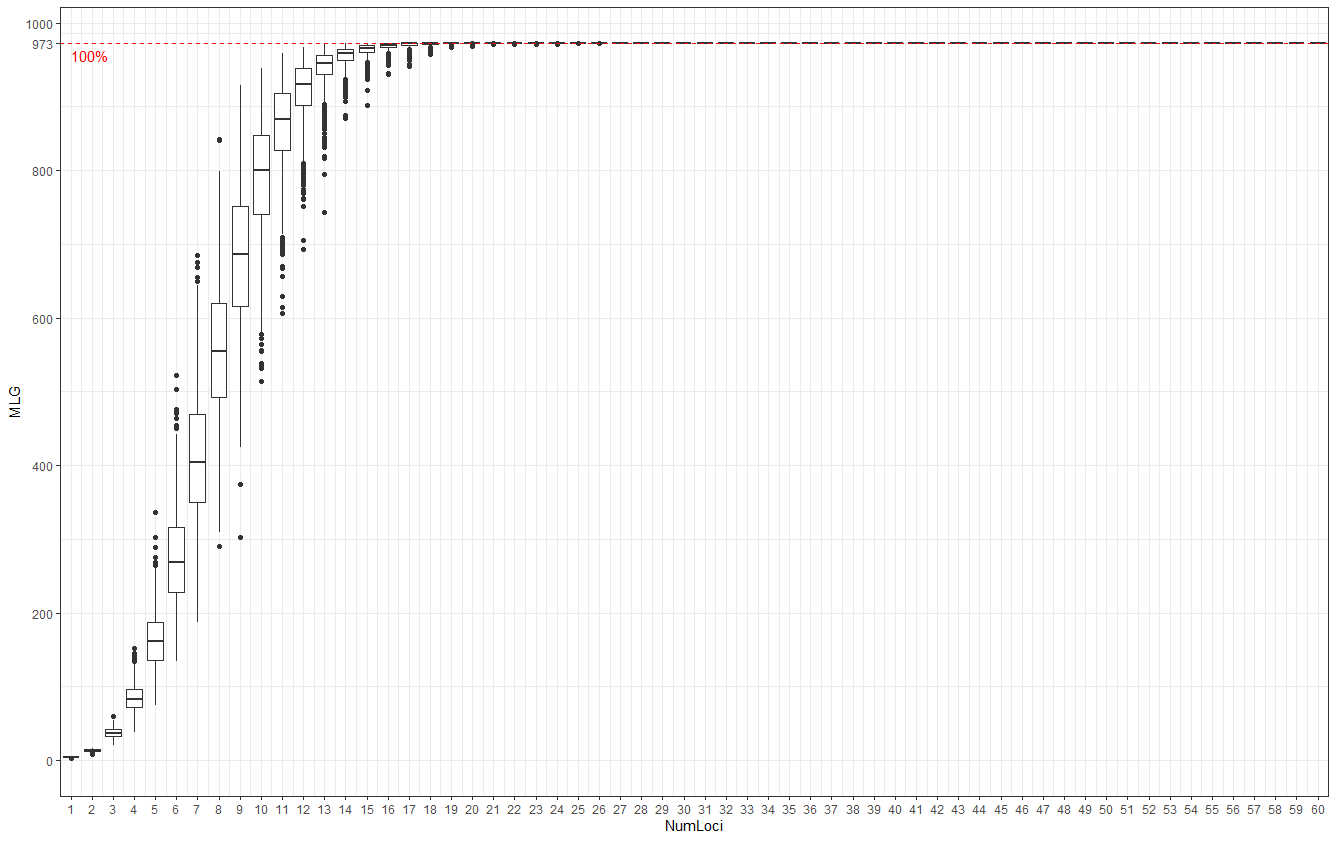


**Fig. S1.** Genotype accumulation curve calculated for the set of 61 polymorphic SNP loci using the total 974 individuals.

| a)  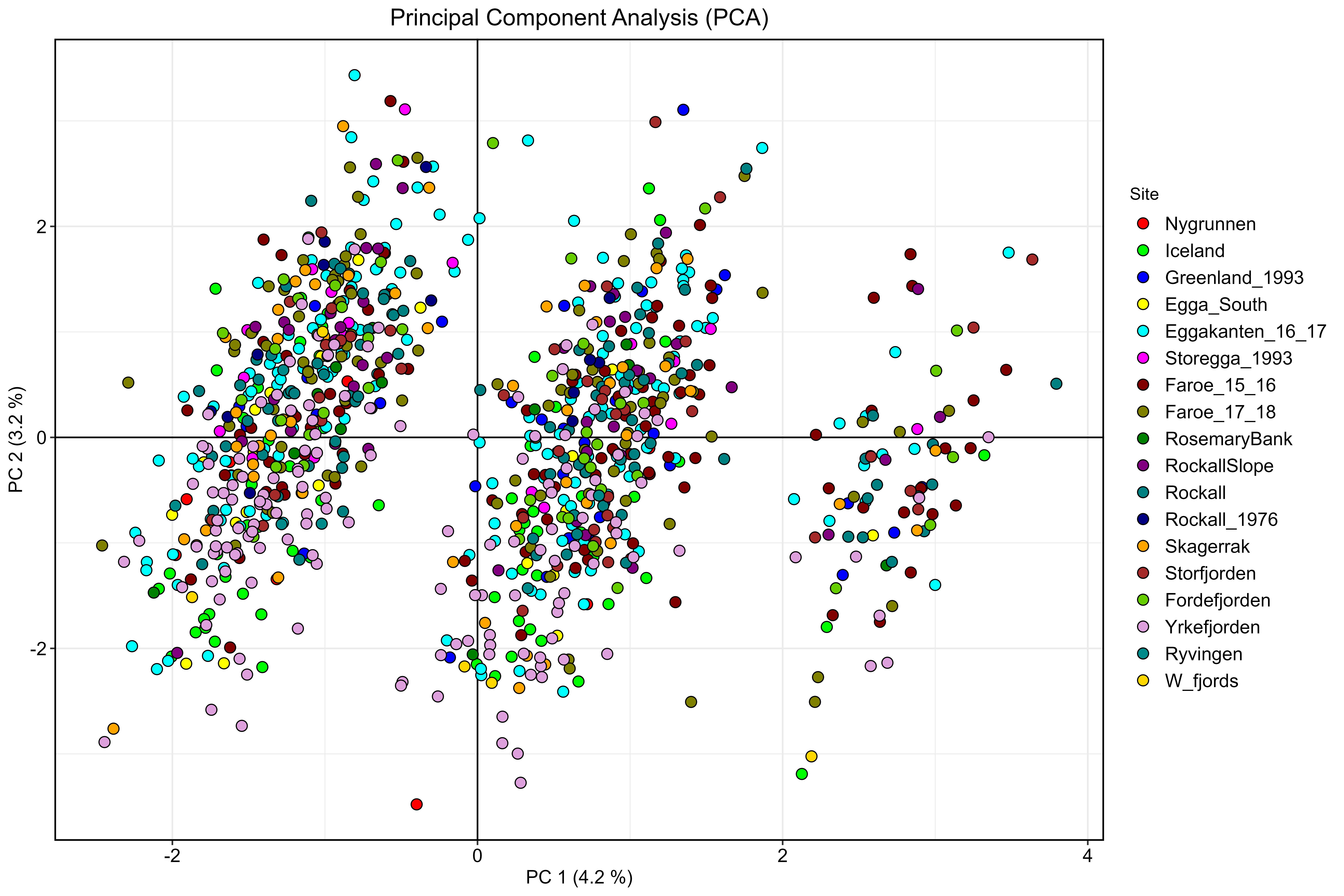 |
| --- |
| b)  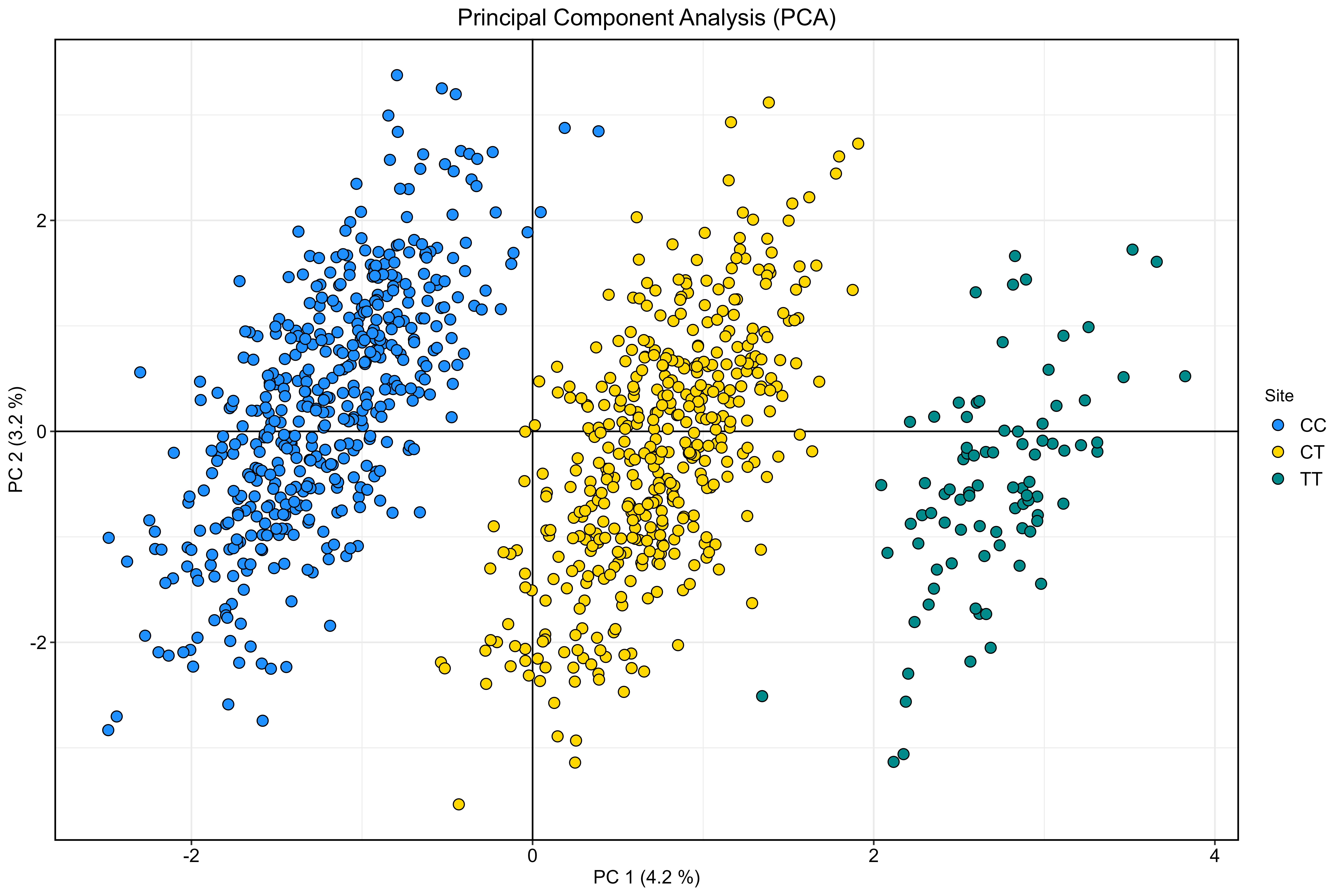 |

**Fig. S2.** PCA biplot of the 974 individuals genotyped at 61 loci (a). The three-stripped clustering is driven by loci Mdy033 and Mdy035 with individuals occupying the central stripe being heterozygotes (CT) whereas the flanking striations host the CC and TT homozygotes, respectively (b).

| a)  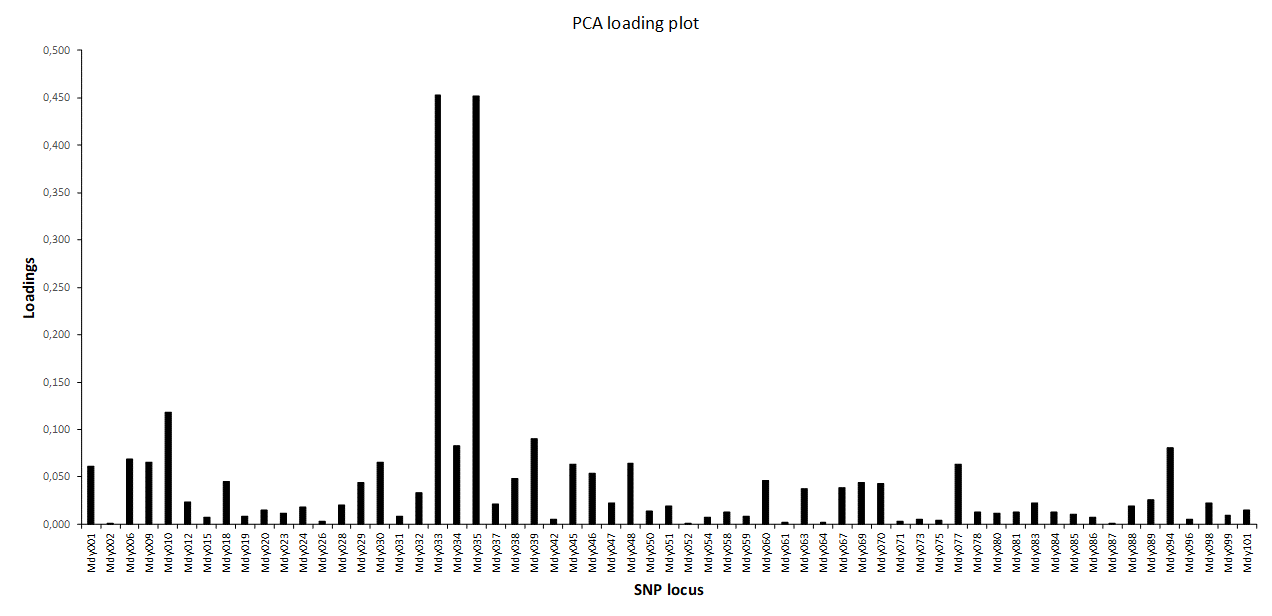 |
| --- |
| b)  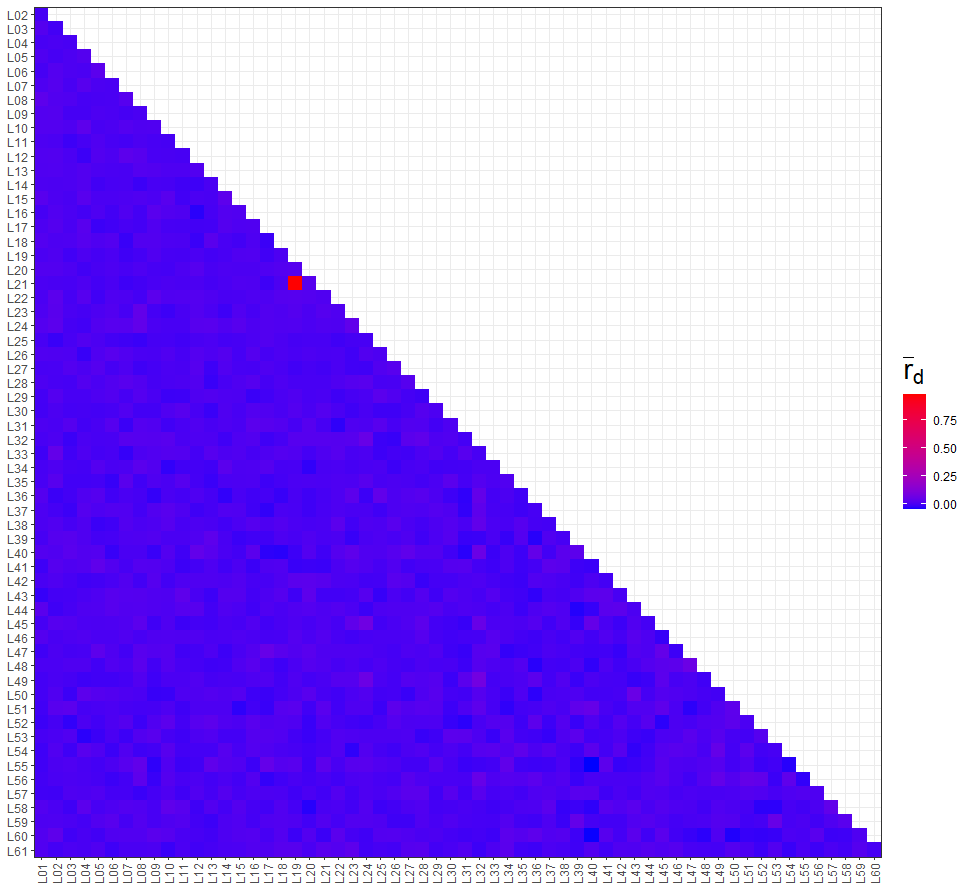 |

**Fig. S3.** PCA loadings calculated for the set of 61 polymorphic SNP loci (a), and heatmap of standardized index of association showing the linkage between loci Mdy033 and Mdy035 (b).


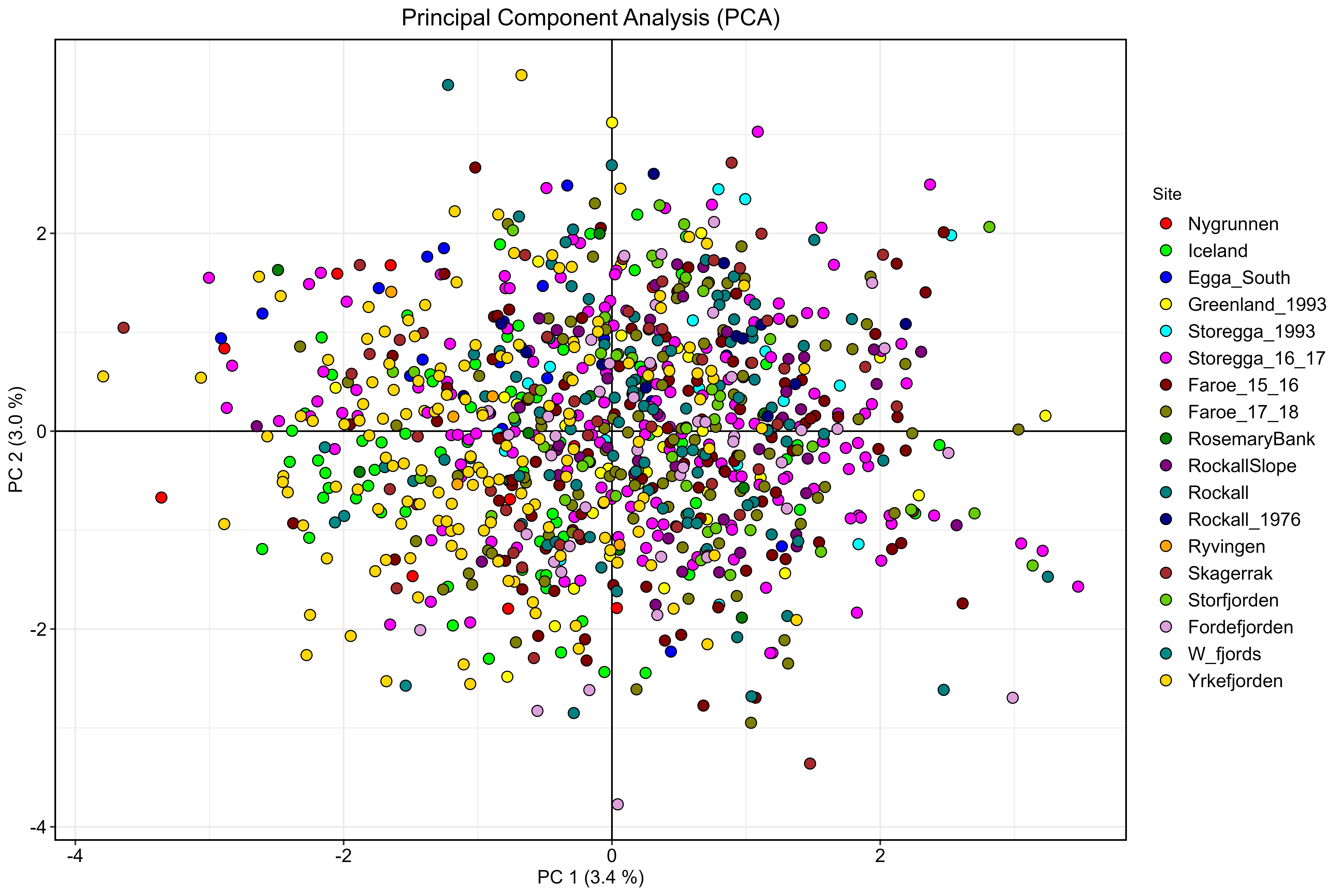


**Fig. S4.** PCA biplot of the 974 individuals after genotyped at 60 loci following LD pruning.

| a)  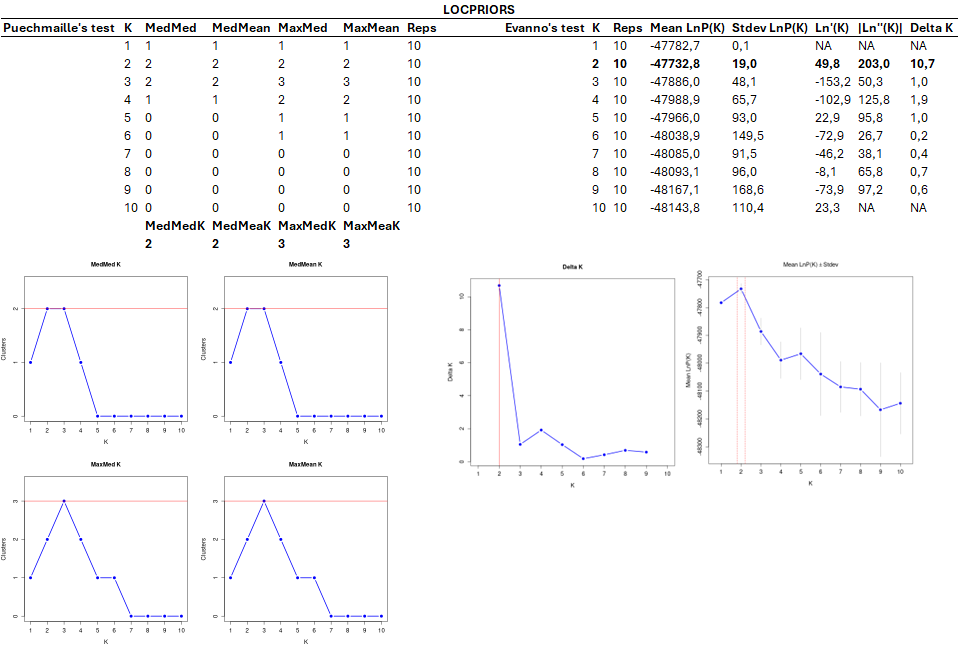 |
| --- |

| b)  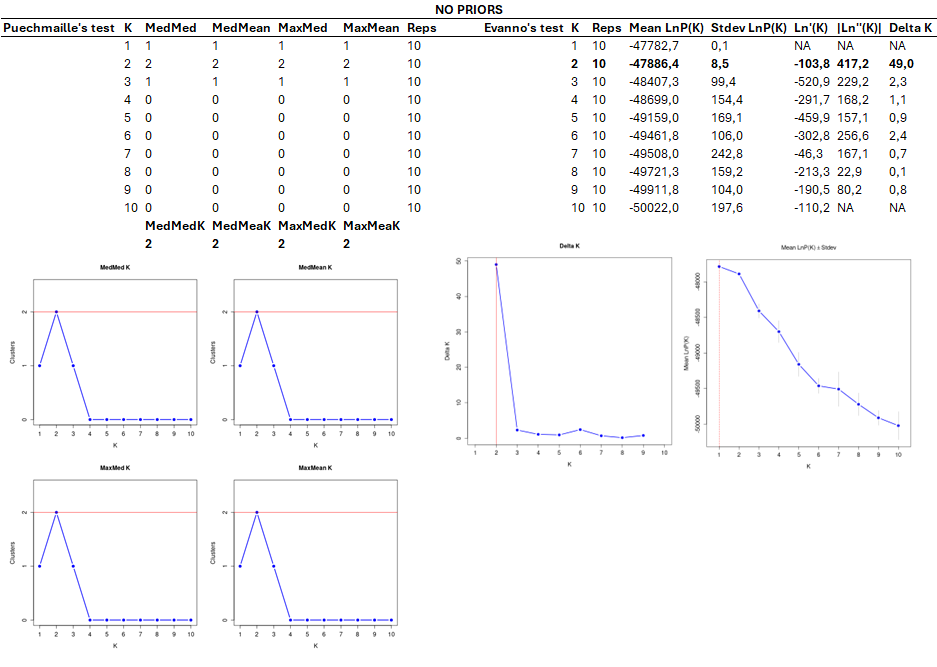 |
| --- |

**Fig. S5.** Output of STRUCTURE with (a) and without (b) using LOC PRIORS using both Puechmaille and Evanno Methods.

**TABLES**

**Table S1.** SNP amplification: primers and distribution of loci in multiplex.

| **Multiplex** | **Locus** | **1st-PCRP** | **2nd-PCRP** | **UEP_SEQ** |
| --- | --- | --- | --- | --- |
| M1 | Mdy_080 | ACGTTGGATGAGTATGGGCAATGAAGGACG | ACGTTGGATGGAAAACGTCCATGGTCCAAC | CCAACATTCCAACAGCA |
| M1 | Mdy_068 | ACGTTGGATGTGTAACTTGTCAGGGATCGG | ACGTTGGATGACACCCCAACAACCAATCAC | GCCCGTGAAATACAAGA |
| M1 | Mdy_023 | ACGTTGGATGGGATCGAGTCATCACGTTAC | ACGTTGGATGCCATGTATAAGGGAACGCAG | AGGGAACGCAGGTATAT |
| M1 | Mdy_032 | ACGTTGGATGCAAAAAGGAACAACCTCTGG | ACGTTGGATGGGCAAACATTGACAGACAGC | aGTTACTGTCAAATGCCG |
| M1 | Mdy_027 | ACGTTGGATGTGCAGGTGGAGGGCTGAGG | ACGTTGGATGGCCCCCTCCCTCATTACAA | TCCCTCATTACAACAACAC |
| M1 | Mdy_025 | ACGTTGGATGTCAGCCAGGTGAGGGAGAG | ACGTTGGATGAGAGGAGAACATGGACCT | AACATGGACCTCCTCCTCC |
| M1 | Mdy_034 | ACGTTGGATGCCTTCCATAGGCTTAGCTTG | ACGTTGGATGAAGGTGTACACCACGAGAAC | caCACGAGAACAAAAACGA |
| M1 | Mdy_056 | ACGTTGGATGCCACTAACCTGCCCAATCTG | ACGTTGGATGGTAGGAGATGCGCTCAACAG | CGGGTTCTGCGAATATAAA |
| M1 | Mdy_078 | ACGTTGGATGCCTGCTCTGTGCCAGGTTT | ACGTTGGATGTATTAATGCTGGAGCCCCTG | CCTCAGTAAAACACTTCCAG |
| M1 | Mdy_096 | ACGTTGGATGTTTGGCTGGGAGTCAAACAC | ACGTTGGATGGAATATAGTAAGGTAACCA | gGTAAGGTAACCAAGCAGTG |
| M1 | Mdy_070 | ACGTTGGATGTGGCCAGTGTTTGATCTGTG | ACGTTGGATGCTAACCTCCGTTTGTAAGCC | caGACAGGCTATGAGAAGAG |
| M1 | Mdy_098 | ACGTTGGATGAGAGAAGCCCCATTTCTTCC | ACGTTGGATGCCTTTGAAAAGGGCTTTCGC | ttCACCACTTTCAGACAAAAC |
| M1 | Mdy_031 | ACGTTGGATGGTACGGTTACATACCGCAGT | ACGTTGGATGGAAGACAGCACTTAACTGAC | TAACCTTGGTGACCATGTAAA |
| M1 | Mdy_029 | ACGTTGGATGTGGATCACAGCAACAGTCAG | ACGTTGGATGATTGGCTGTTCGAACCCAGA | cCTGTTCGAACCCAGATTCTTT |
| M1 | Mdy_001 | ACGTTGGATGGGTCACCTCATGATTTAAC | ACGTTGGATGTGCAAAGCTGTTCGACATGA | agGTTGAGCTGTATTGACCATA |
| M1 | Mdy_088 | ACGTTGGATGGGATCAATGAACCGAACCAC | ACGTTGGATGGCCTAAATAAAGGAAGTCTG | tCACTAAAGTCAAACGCTGACTA |
| M1 | Mdy_067 | ACGTTGGATGGCTCAAATTGCCATGAGATT | ACGTTGGATGTGCTTTCTGGGTCCGAATAG | gTTTTACGTAACAGAGCACTAAG |
| M1 | Mdy_085 | ACGTTGGATGTCGAGCGAGGAGCTCCCTAT | ACGTTGGATGGAGAATGTTACCAGGCCTTC | tgCCTGCCTTACCGCTCCAACACA |
| M1 | Mdy_015 | ACGTTGGATGCTCCTATATGATCATGGCCC | ACGTTGGATGTTCTCCAGGTCAAAGGTCAG | AAGGTCAGGAAGGTCAAGGTGAT |
| M1 | Mdy_045 | ACGTTGGATGCGTCCGTCGCTTTTATTGTG | ACGTTGGATGCACTGACTGATATTGAAAGGC | ggTTGAAAGGCAATCAACGCCCCA |
| M1 | Mdy_035 | ACGTTGGATGGAAGTCAAACTGTCTGAATG | ACGTTGGATGATAAATTGCCTACTCTAGC | tTTGCCTACTCTAGCTTTAAATTAC |
| M1 | Mdy_103 | ACGTTGGATGCACAGATTCTTAAGCTAGCAG | ACGTTGGATGCCCCACTCTACTATATGGTC | tGCTCTGACCCAGTTCTGTTGTTGC |
| M1 | Mdy_050 | ACGTTGGATGCCAAACAGCAAAATGAATGCG | ACGTTGGATGAGAGGGAGAGAATGTGCTTG | aTAGTGATAATGTCAGTCTGATAAT |
| M1 | Mdy_020 | ACGTTGGATGTGCAGCCACTTTGTATTAGG | ACGTTGGATGTGCGTGAGGTAAAACCATTG | TGTAGAGAACACATTGAAATAGATG |
| M1 | Mdy_009 | ACGTTGGATGGGTTCTCGTGGAGCTGATG | ACGTTGGATGGGAAGAGAGCTCTGGTCTAC | ggCTACATGGTCCCATGGCAGCGTCC |
| M1 | Mdy_005 | ACGTTGGATGGGTACTGACAGAAGGAAAGC | ACGTTGGATGATATCTCCCCAGAACAACAG | TATCTCCCCAGAACAACAGAGTGAGA |
| M1 | Mdy_091 | ACGTTGGATGACTTGATATTGGCAGGGCTC | ACGTTGGATGGTGTCCAGGAGTTTGTCAAG | ggGGAGTTTGTCAAGTGGTCCGTGAA |
| M1 | Mdy_086 | ACGTTGGATGTTCTCCACAGACTAGTTATG | ACGTTGGATGGGATGGTCAAGTGTCTAAAG | AAAACCATCAGCCCAATGATTTCTTTC |
|  |  |  |  |  |
| M2 | Mdy_073 | ACGTTGGATGTGCATATGCACACAGTACCG | ACGTTGGATGCAGCTCTGCTCTCACACTC | CTCTCACACTCCAAACT |
| M2 | Mdy_064 | ACGTTGGATGAATCGCTTGGATGTACGGTG | ACGTTGGATGCATGCTAAATGCTTCCCGAG | GTGGCACACACACTAAT |
| M2 | Mdy_054 | ACGTTGGATGGCAGGATGAGAATCAAACAC | ACGTTGGATGGCACAATAAAGAGTCGACCG | AGTCAGAATGACGCAGA |
| M2 | Mdy_059 | ACGTTGGATGACGCGCTGTTACACACAAAG | ACGTTGGATGATAGCTACGTGCTAGCTGTG | ACGCATACACTCTTCAGT |
| M2 | Mdy_026 | ACGTTGGATGACCATGGCGTTTATGCAGTC | ACGTTGGATGAAGAAGACGGTCTCCTTCAG | GGAGAAGAAGATCAGCAG |
| M2 | Mdy_087 | ACGTTGGATGACTCAGTTCCCTTAAGGTGG | ACGTTGGATGCTAGCTGTGGAAAGCAGAAC | CGTTCTTACACTACAGTGG |
| M2 | Mdy_101 | ACGTTGGATGATTGGGTTGGGTTTCTTCTC | ACGTTGGATGAACCAGAACGGTCCTACTTC | cctGTCCTACTTCAACCGGC |
| M2 | Mdy_099 | ACGTTGGATGACGTGTAAGGAGCACGTTTG | ACGTTGGATGATCAATTTTGGCCTGCATCG | ctCGATGCACGGCACTACAA |
| M2 | Mdy_019 | ACGTTGGATGACTCTCTCCTGTAGGTCAGC | ACGTTGGATGTCTCTCTATTTCAGACGAGG | GACGAGGTAGAGCGTTTCCA |
| M2 | Mdy_042 | ACGTTGGATGTGCTTAGCGCAAACATGACC | ACGTTGGATGCAACTTTTGCACTCTCAAGG | ccgaACTCTCAAGGCATTCCT |
| M2 | Mdy_040 | ACGTTGGATGAACTGAAAGTCACGAGTCGC | ACGTTGGATGGAGTCAGCCAATCAACACGC | cCGCTGGGCTGGTGTCAGCAA |
| M2 | Mdy_002 | ACGTTGGATGTGGCCAGGTCCATGTCCAG | ACGTTGGATGTCAACAATCACACGGTGCAG | ggGATGCGGAACCTGACGTGG |
| M2 | Mdy_012 | ACGTTGGATGGCCGTACTAGTAGGTACATC | ACGTTGGATGAGCTCAGAATCACTGAAGCG | ccccATACATGCAAGCACCGGA |
| M2 | Mdy_063 | ACGTTGGATGGTTCAGCAGATTCTAGACCG | ACGTTGGATGTAACATGCATTAGGGCGCTC | GCTCGTAAGATCTAGGTTTTGA |
| M2 | Mdy_102 | ACGTTGGATGCTGCTCAACTGTGAGTAACG | ACGTTGGATGTAGCGTCTAGCCCTTCAAAC | ggggCCCAGTGCCCCCCTCTCTC |
| M2 | Mdy_028 | ACGTTGGATGTTTGAACAGGTCCCGGTTAG | ACGTTGGATGTGTGCCAAAGCAAGAATGTC | GTGTACACAAAAACATCAGTAAA |
| M2 | Mdy_090 | ACGTTGGATGATGTCCGCAGCCTTATAACC | ACGTTGGATGACATGCATGTCACTGAGGAG | gagaGGGTGTAGGGTCAGGCCAT |
| M2 | Mdy_051 | ACGTTGGATGAAAGCAGATTCCCCGGTTAG | ACGTTGGATGCGGAAGAAAAACCCCATGTC | TGTCAACTTTACTCACAAATAAAA |
| M2 | Mdy_074 | ACGTTGGATGTCTTTCCAAACGACTGTCCC | ACGTTGGATGCCACAGCACCACTGAACTTC | ggggCTGAACTTCAGCTACACGGG |
| M2 | Mdy_049 | ACGTTGGATGAGCTGCTCCTCCTACCTCCT | ACGTTGGATGTCCTACTTCCACGTCACCTG | gtCTCAGAGGGAGGGGCTCCTGTC |
| M2 | Mdy_030 | ACGTTGGATGTGTGTGAACGCCCGGACATT | ACGTTGGATGCTTTACGCTTCTGTGCAGGG | ccttAGGGGATATGTCATCCATCTC |
| M2 | Mdy_046 | ACGTTGGATGCCATTTTGACATGTAGGCAC | ACGTTGGATGTTCAAATTCACGGGCAGTCG | TTATCTTTGGATAAAATAACCATCA |
| M2 | Mdy_033 | ACGTTGGATGGAAGTCAAACTGTCTGAATG | ACGTTGGATGATAAATTGCCTACTCTAGC | cctcGCCTACTCTAGCTTTAAATTAC |
| M2 | Mdy_077 | ACGTTGGATGCCTCGTCACATTGTGGTTTG | ACGTTGGATGGCCTGAAACCCCGAAAAAAC | ggacACCCAGGTATAAATTAGGCTTT |
| M2 | Mdy_048 | ACGTTGGATGGCTCCATTCTTCATAGTGGG | ACGTTGGATGTGCAGGGTTAAACCATCGTC | ggtaACAGAAGTGTGATTATTCATTT |
| M2 | Mdy_094 | ACGTTGGATGTCCACCAAATAGCATGGTCC | ACGTTGGATGGCGGCATCATCAACAATACC | tcccATACCAATTAGCATTAGCATACT |
| M2 | Mdy_037 | ACGTTGGATGTGCTGTAGTTCCTTGGGAAA | ACGTTGGATGCCACTGTATCATTATTTGGTC | cCTGTATCATTATTTGGTCATTATTTC |
|  |  |  |  |  |
| M3 | Mdy_038 | ACGTTGGATGCCACTTCAGACTTCATCTGG | ACGTTGGATGGGTCCAAACGCTCTTCTCTC | CTCTTCTCTCACAGACC |
| M3 | Mdy_071 | ACGTTGGATGACTCCATTTTCCACACGGAC | ACGTTGGATGAAGGTCACCTGCCATTAACG | CAAATAAACACCGGCTC |
| M3 | Mdy_058 | ACGTTGGATGTGAGGGTGTTTGGGGTTGTG | ACGTTGGATGCACATCTTTATTTCTCGCAG | AAGATGAATGCACTGCC |
| M3 | Mdy_021 | ACGTTGGATGGAGCACCACATCCTATTTAG | ACGTTGGATGGTTGAAAGGTCTGTTATTTG | TTGTGTTGGTTGAAGGG |
| M3 | Mdy_069 | ACGTTGGATGAGCGGCGGGGTTCCCTGTTT | ACGTTGGATGCCTCCATACCAAACCAACTC | AAACCATTGACCGCGTAG |
| M3 | Mdy_041 | ACGTTGGATGGACACAGCAGAGACCTTATG | ACGTTGGATGTCAACGTTACGTACTGCACC | TGCACCTTTAAACTCAAAC |
| M3 | Mdy_004 | ACGTTGGATGATTGGAAACAGACGTTGGGC | ACGTTGGATGAGGACACACCACAAGAGATG | ACAAGAGATGTGTGCGCCC |
| M3 | Mdy_084 | ACGTTGGATGCCTGACACCTCCAATCATTC | ACGTTGGATGAGCAACCTGGAAGGCCTTTC | agCTGAACGAATGCGATAC |
| M3 | Mdy_039 | ACGTTGGATGAATTCCACCTCTCTCCTGTC | ACGTTGGATGAATCACTGCTGACTGACGAG | aAGGAAGTCCAGGAGATGG |
| M3 | Mdy_083 | ACGTTGGATGCAAAAGAGTCTGTTCACTGC | ACGTTGGATGGAGCAACGTCTCTTTTTTGG | TGTTATGAATCATCATCCAC |
| M3 | Mdy_092 | ACGTTGGATGATGCTCGGTTTCCTCCTTTG | ACGTTGGATGAAGACCCACGAACAAACAGC | GAACAAACAGCAACTGAAGAC |
| M3 | Mdy_007 | ACGTTGGATGCCACGCGCAGACATTTATAC | ACGTTGGATGTTACTCAAGGTGGCCATAGG | GTATGTGTGTTAATAATCGGA |
| M3 | Mdy_060 | ACGTTGGATGAGATCTATGGTAACCAATG | ACGTTGGATGACAAGGTCGACCATTGAAGG | cATTAACCACATTCTCAGTATT |
| M3 | Mdy_010 | ACGTTGGATGGGTGTGTATGTATGTGTGGC | ACGTTGGATGCGCTGATTAACTTCCTAAAG | AAAGTAGTTTTTGGAACAAAAT |
| M3 | Mdy_052 | ACGTTGGATGCGTCTGATGTTGCATTGTCC | ACGTTGGATGTTCAATAGGAACTGGAAGGG | ggGGATCTTGAAGATAAAGAGG |
| M3 | Mdy_061 | ACGTTGGATGCACGTCATGGGTTTAAACCG | ACGTTGGATGGAGGATACCCACATGAACAC | CACGGAGAACATACAAACTCACA |
| M3 | Mdy_081 | ACGTTGGATGGGTTGCAAAGTAAGACATTTC | ACGTTGGATGTCACCCTGGGTTTAGTACAC | GTACACAGCAGCATCTGAGAAAG |
| M3 | Mdy_089 | ACGTTGGATGATGTTACGCAACGACCACAC | ACGTTGGATGCATGTGTTGTGTCCGCTAAG | ccCTAAGCCTCCATAACGTCTGGC |
| M3 | Mdy_062 | ACGTTGGATGTCTGAGTTGCGTGCTGTTTG | ACGTTGGATGGGGAATTCTCTCCCAAACAT | ggtCCAACATCATGGATAGTCCAC |
| M3 | Mdy_047 | ACGTTGGATGGCTGTTGTCAAAATGGTGGG | ACGTTGGATGGCTGCCAACACAGATAAGAC | AAGACAGATATGTTATAAGGAGAT |
| M3 | Mdy_065 | ACGTTGGATGATTGAGGGCAGGTAAAGTGG | ACGTTGGATGGCATGCGTGTACACATTCAG | tTACACATTCAGATCATGTATGAAC |
| M3 | Mdy_024 | ACGTTGGATGATGTGTCACACACATGGGTC | ACGTTGGATGCAGTCCCAGGAGATTTGTGC | ATGATAGAATGTTCTCGTGTATATT |
| M3 | Mdy_016 | ACGTTGGATGAATACTGTGCACTGGCTACC | ACGTTGGATGGCAACTAGCCGTTGAAATCC | cccCCAAAAACATGACGCAGTTTCAC |
| M3 | Mdy_006 | ACGTTGGATGGTCCCAACCCATTGTTATGC | ACGTTGGATGACAGGTTAGTCTCCCCAAAC | gCCAAACAGCCGTGTTTCTGGGAAAG |
| M3 | Mdy_075 | ACGTTGGATGAGATTTGGACCGTGAATTCG | ACGTTGGATGTACCCTGAGTACAGAGATAC | tTACAGAGATACAAATAAGATGAAAG |
| M3 | Mdy_018 | ACGTTGGATGGGGACGTACCAGGAATTTTG | ACGTTGGATGCTACCCTGTAGGCATTCTTG | ctTAAGATGTCCAACACCTAGATGACC |

**Table S2.** Outlier detection analyses: outcome from BayeScan and Arlequin. None of the loci displayed deviation from neutral expectations.

|  | **BayeScan** | | | **Arlequin** | | |
| --- | --- | --- | --- | --- | --- | --- |
| **Locus** | **log_10_(PO)** | **qval** | ***F*_ST_** | **H_O_** | ***F*_ST_** | **P-val** |
| Mdy001 | -1.069 | 0.803 | 0.017 | 0.482 | 0.005 | 0.487 |
| Mdy002 | -1.107 | 0.813 | 0.018 | 0.487 | 0.028 | 0.052 |
| Mdy006 | -0.415 | 0.587 | 0.014 | 0.500 | -0.001 | 0.302 |
| Mdy009 | -1.288 | 0.862 | 0.018 | 0.490 | 0.012 | 0.333 |
| Mdy010 | -1.217 | 0.843 | 0.018 | 0.500 | 0.013 | 0.291 |
| Mdy012 | -1.127 | 0.816 | 0.017 | 0.459 | 0.009 | 0.419 |
| Mdy015 | -0.924 | 0.745 | 0.017 | 0.495 | 0.007 | 0.480 |
| Mdy018 | 0.002 | 0.447 | 0.011 | 0.485 | -0.003 | 0.256 |
| Mdy019 | -1.200 | 0.837 | 0.017 | 0.454 | 0.013 | 0.312 |
| Mdy020 | -0.921 | 0.738 | 0.017 | 0.500 | 0.001 | 0.358 |
| Mdy023 | -0.779 | 0.650 | 0.016 | 0.373 | 0.000 | 0.330 |
| Mdy024 | -0.857 | 0.712 | 0.017 | 0.316 | 0.002 | 0.376 |
| Mdy026 | -0.916 | 0.722 | 0.017 | 0.481 | 0.004 | 0.431 |
| Mdy028 | -1.032 | 0.788 | 0.017 | 0.497 | 0.004 | 0.435 |
| Mdy029 | 0.143 | 0.418 | 0.010 | 0.458 | -0.003 | 0.256 |
| Mdy030 | -1.263 | 0.859 | 0.018 | 0.501 | 0.019 | 0.169 |
| Mdy031 | -0.939 | 0.752 | 0.017 | 0.273 | 0.003 | 0.394 |
| Mdy032 | -1.001 | 0.779 | 0.017 | 0.498 | 0.003 | 0.427 |
| Mdy033 | -1.218 | 0.845 | 0.018 | 0.426 | 0.009 | 0.405 |
| Mdy034 | -0.401 | 0.571 | 0.014 | 0.412 | -0.002 | 0.290 |
| Mdy035 | -1.224 | 0.849 | 0.018 | 0.426 | 0.010 | 0.394 |
| Mdy037 | -1.214 | 0.841 | 0.018 | na | na | na |
| Mdy038 | -0.700 | 0.633 | 0.016 | 0.500 | 0.000 | 0.331 |
| Mdy039 | -1.228 | 0.851 | 0.018 | 0.421 | 0.010 | 0.394 |
| Mdy042 | -0.185 | 0.498 | 0.013 | 0.376 | -0.002 | 0.282 |
| Mdy045 | -1.244 | 0.858 | 0.018 | 0.458 | 0.014 | 0.272 |
| Mdy046 | -1.222 | 0.847 | 0.018 | 0.409 | 0.020 | 0.144 |
| Mdy047 | -0.082 | 0.472 | 0.012 | 0.478 | -0.003 | 0.251 |
| Mdy048 | -1.057 | 0.799 | 0.017 | 0.498 | 0.003 | 0.419 |
| Mdy050 | -0.348 | 0.550 | 0.014 | 0.382 | 0.001 | 0.364 |
| Mdy051 | -0.447 | 0.614 | 0.015 | 0.214 | -0.001 | 0.308 |
| Mdy052 | -1.187 | 0.832 | 0.017 | 0.312 | 0.011 | 0.356 |
| Mdy054 | -0.854 | 0.703 | 0.016 | 0.324 | 0.000 | 0.337 |
| Mdy058 | -0.300 | 0.526 | 0.014 | 0.224 | -0.002 | 0.284 |
| Mdy059 | -0.832 | 0.680 | 0.016 | 0.383 | 0.002 | 0.379 |
| Mdy060 | -1.334 | 0.864 | 0.018 | 0.187 | 0.022 | 0.124 |
| Mdy061 | -1.244 | 0.858 | 0.017 | 0.316 | 0.018 | 0.204 |
| Mdy063 | -1.137 | 0.824 | 0.017 | 0.350 | 0.008 | 0.423 |
| Mdy064 | -1.086 | 0.810 | 0.017 | 0.229 | 0.006 | 0.497 |
| Mdy067 | -1.196 | 0.835 | 0.017 | 0.171 | 0.020 | 0.159 |
| Mdy069 | -0.445 | 0.602 | 0.015 | 0.342 | -0.001 | 0.298 |
| Mdy070 | -1.036 | 0.792 | 0.017 | 0.250 | 0.009 | 0.419 |
| Mdy071 | -1.017 | 0.783 | 0.017 | 0.186 | 0.002 | 0.398 |
| Mdy073 | -0.951 | 0.763 | 0.017 | 0.270 | 0.002 | 0.378 |
| Mdy075 | -0.960 | 0.769 | 0.017 | 0.197 | 0.002 | 0.372 |
| Mdy077 | -1.050 | 0.796 | 0.017 | 0.366 | 0.010 | 0.388 |
| Mdy078 | -1.265 | 0.861 | 0.018 | 0.185 | 0.017 | 0.206 |
| Mdy080 | -1.236 | 0.854 | 0.018 | 0.262 | 0.018 | 0.199 |
| Mdy081 | -0.943 | 0.758 | 0.017 | 0.198 | 0.007 | 0.462 |
| Mdy083 | -1.229 | 0.853 | 0.018 | 0.184 | 0.018 | 0.184 |
| Mdy084 | -1.206 | 0.839 | 0.018 | 0.138 | 0.009 | 0.411 |
| Mdy085 | -1.158 | 0.827 | 0.018 | 0.244 | 0.009 | 0.405 |
| Mdy086 | -1.079 | 0.806 | 0.017 | 0.259 | 0.003 | 0.411 |
| Mdy087 | -0.832 | 0.680 | 0.016 | 0.185 | 0.006 | 0.495 |
| Mdy088 | -0.919 | 0.730 | 0.017 | 0.136 | 0.000 | 0.342 |
| Mdy089 | -0.978 | 0.774 | 0.017 | 0.285 | 0.003 | 0.417 |
| Mdy094 | -1.185 | 0.830 | 0.018 | 0.407 | 0.009 | 0.413 |
| Mdy096 | 0.134 | 0.421 | 0.010 | 0.114 | -0.004 | 0.231 |
| Mdy098 | -1.129 | 0.819 | 0.017 | 0.333 | 0.003 | 0.416 |
| Mdy099 | -0.840 | 0.692 | 0.016 | na | na | na |
| Mdy101 | -1.132 | 0.822 | 0.017 | 0.399 | 0.005 | 0.472 |

**Table S3.** Clines analyses: Loci showing significant clines with delta frequency >2, where p-val depicts the significance value of the linear regression; r^2^ is the measurement of the fit to the linear model; minFreq and maxFreq are the minimum and maximum observed frequency of an allele in all samples, respectively and ΔFreq is the absolute difference between minFreq and maxFreq. Permutation tests over the loci that adhered to the cline pattern and corresponding p-value. Boldface type depicts p-values significantly different from zero.

|  | **Cline analyses** | | | | | **Permutation tests** |
| --- | --- | --- | --- | --- | --- | --- |
| **Locus** | **p-val** | **r^2^** | **maxAllFreq** | **minAllFreq** | **ΔFreq** | **P-value** |
| Mdy010 | **0.038** | 0.539 | 0.657 | 0.292 | 0.365 | **0.0087** |
| Mdy019 | **0.002** | 0.809 | 0.750 | 0.500 | 0.250 | **0.0054** |
| Mdy028 | **0.019** | 0.629 | 0.625 | 0.269 | 0.356 | **0.0298** |
| Mdy030 | **0.029** | 0.574 | 0.694 | 0.318 | 0.376 | **0.0424** |
| Mdy052 | **0.020** | 0.621 | 0.909 | 0.696 | 0.213 | **0.0147** |
| Mdy080 | **0.031** | 0.565 | 0.958 | 0.731 | 0.227 | **0.0030** |
| Mdy085 | **0.017** | 0.640 | 0.962 | 0.667 | 0.295 | 0.0654 |
| Mdy101 | **0.044** | 0.517 | 0.816 | 0.580 | 0.236 | **0.0312** |
